# Supplementary material for: Drug delivery process simulation—Quantifying the conformation dynamics of paclitaxel and cremophor EL
Source: PLoS One. 2025 May 12;20(5):e0313813. doi: 10.1371/journal.pone.0313813 (PMC12068633; doi:10.1371/journal.pone.0313813)
Supplement: S1 File — Taxol micelle analysis (S1_File.pdf): more detailed analysis of the time evolution of PTX and CrEL interactions in the Taxol micelle is presented. This includes mean square displacements for PTX and CrEL, plus radial distribution functions illustrating the interactions of PTX with the head and tail groups of the CrEL molecules. (PDF) [file pone.0313813.s001.pdf]

S1 File. Taxol Micelle

Drug Delivery Process Simulation - Quantifying the Conformation Dynamics of Paclitaxel and Cremophor EL

Mafiz Uddin[1*] and Dennis Coombe[2]

[1] Alberta Computational Biochemistry Lab, Edmonton, AB, Canada
[2] Computer Modelling Group, Calgary, AB, Canada

* Corresponding author
E-mail: mafiz.uddin36@gmail.com

Time Evolution of Taxol Micelle

The long time (1000 ns) CG results for Taxol micelles containing 8PTX and 32PTX with and without water approach steady state by this time (no changes over 700-1000 ns). S1 and S2 Tables summarized several basic properties for 8PTX Taxol micelle.

S1 Table. Basic Properties – 8PTX Taxol Micelle

Time (ns)	Volume (nm^3)	Density (kg/m^3)	Potential Energy ($kJ.mol^{-1}$)	LJ (short range) ($kJ.mol^{-1}$)
800	1816.780	896.568	-71.292	-68.113
900	1816.740	896.591	-71.379	-68.155
1000	1816.370	896.772	-71.505	-68.211

S2 Table. Basic Properties - 8PTX Taxol Micelle in Water.

Time *(ns)*	Radius of ME *(nm)*	Volume *(nm^3)*	Density *(kg/m^3)*	Potential Energy *($kJ.mol^{-1}$)*
800	5.810	12645.20	1114.20	-32.267
900	5.800	12642.30	1114.46	-34.278
1000	5.785	12633.30	1115.25	-34.309

Mean Square Displacement

The mean square displacement (MSD) and the self diffusion (D) of the particles are calculated by the GROMACS analysis program "g_msd". The MSD for EOH, PTX and CrEL are shown in S1 to S6 Figures. The diffusion constants are calculated by least squares fitting a straight line (D*t+c) through the MSD(t). An error estimate is given, which is the difference of the diffusion coefficients obtained from fits over the two halves of the fit interval. The important

observation here is that PTX mobility is shown to be highly transient. The results show that the slopes of the MSD lines reduced over time. Here, the MSD lines of CrEL and EOH get clustered and approximately stabilized over time 800-1000ns. Conversely there are some fluctuations in the slopes of the MSD lines of PTX. The PTX MSD lines were not stabilized.

In order to quantify the PTX mobility further, we have monitored the PTX mobility paths at every 20ns MD trajectories. The PTX paths showed some repeated patterns within the CrEL aggregated cluster. The PTX mobility here is mainly controlled by the CrEL local aggregation around the PTX molecules. As CrEL molecules were fully stabilized (very low mobility), they are incapable of stabilizing the oscillatory behaviours of the trapped PTX within the CrEL cavities (here, PTX molecules have no access energy in the equilibrium Taxol system to overcome the local energy barrier within the cavity).

The mean square displacement (MSD) for the Taxol micelles are shown in S1 to S6 Figures. The important observation here is that PTX mobility is shown to be highly transient, and fluctuates significantly over time. Also the slopes of the MSD lines (related to PTX diffusion in the Taxol micelle) are reduced over the time with some fluctuations. Conversely, the MSD lines of CrEL and EOH cluster and stabilize over time 800-1000ns. In summary, PTX paths here show non-stabilized patterns within the CrEL aggregated cluster.

Generally, the high concentration (32PTX) Taxol micelle shows larger MSD oscillations over time as compared with the standard concentration (8PTX) micelle. This is attributed to the reduced ability of available CrEL (200 CrEL molecules) to stabilize the necessary hydrophobic regions locally surrounding each PTX molecule in the high concentration case.

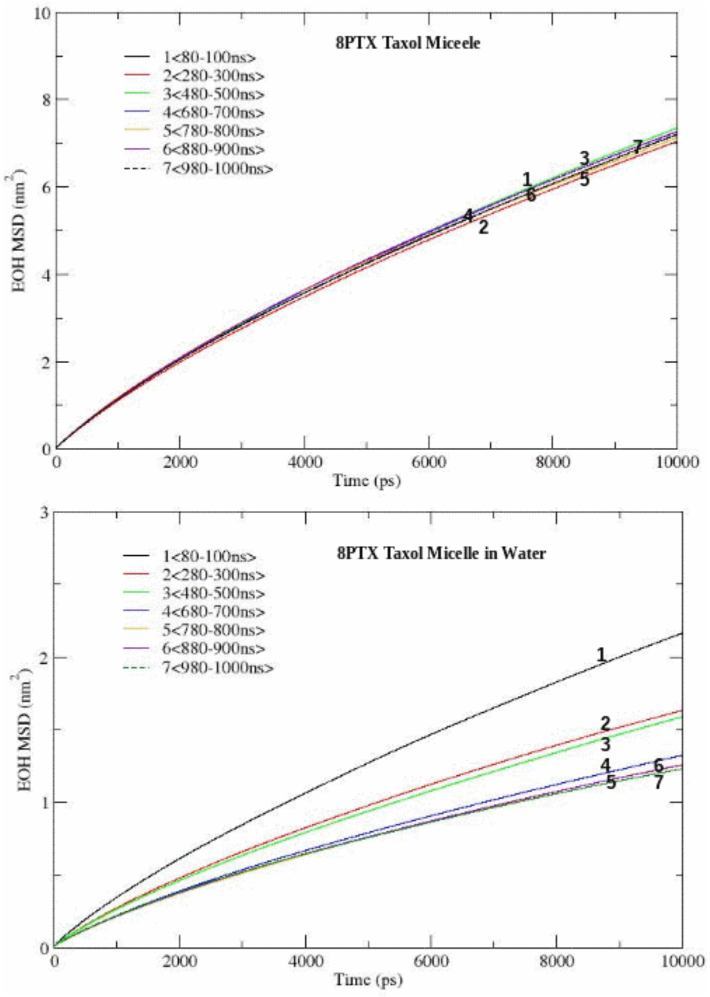

S1 Fig. EOH mean square displacements in 8PTX Taxol micelle with and without water.

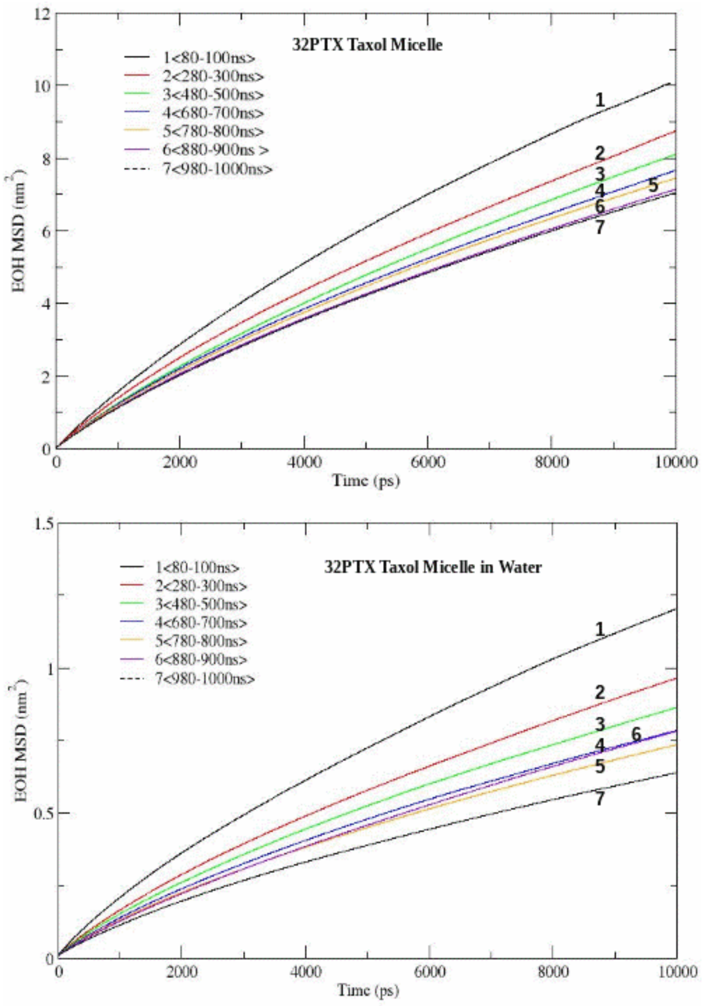

S2 Fig. EOH mean square displacements in 32PTX Taxol micelle without and with water (average over last 20ns MD trajectories).

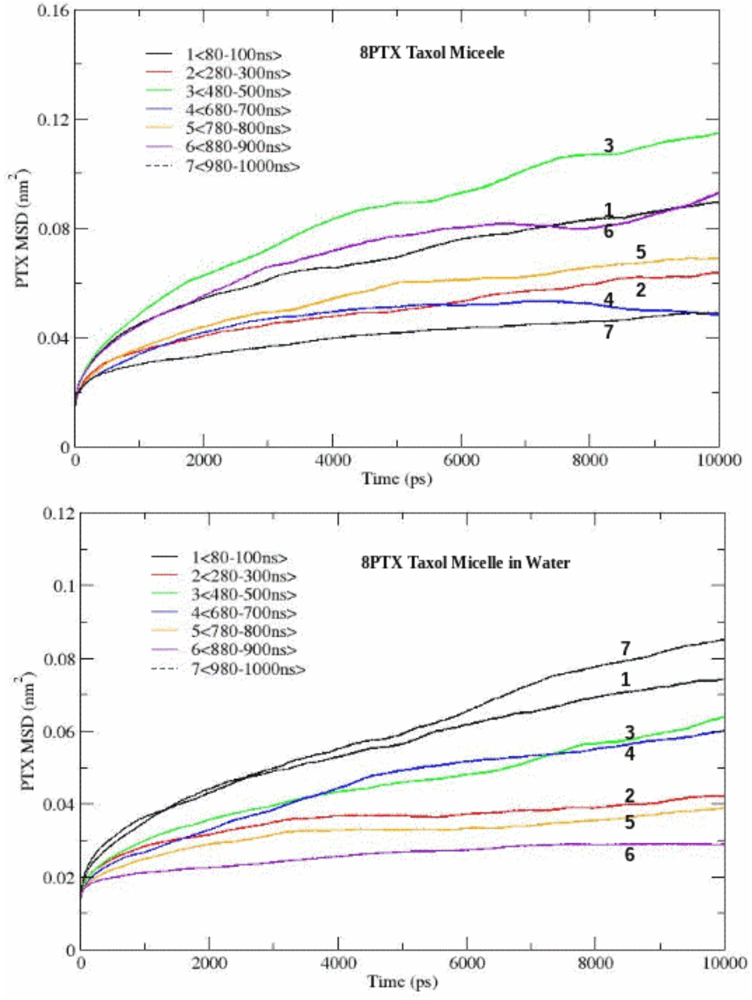

S3 Fig. PTX mean square displacements in 8PTX Taxol micelle with and without water.

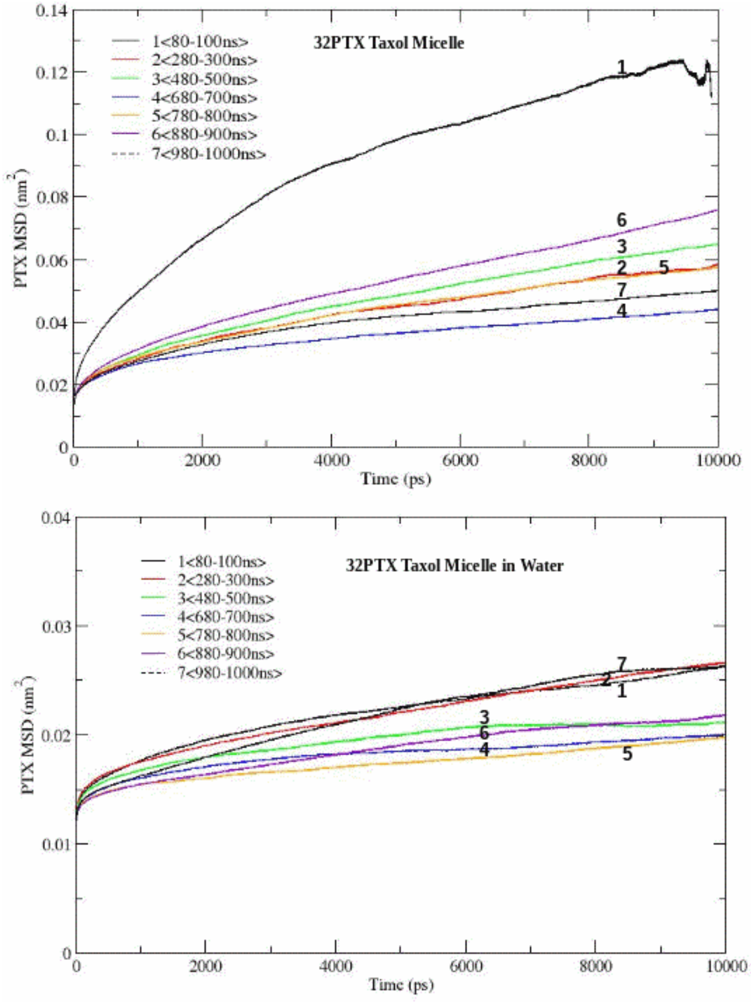

S4 Fig. PTX mean square displacements in 32PTX Taxol micelle without and with water (average over last 20ns MD trajectories).

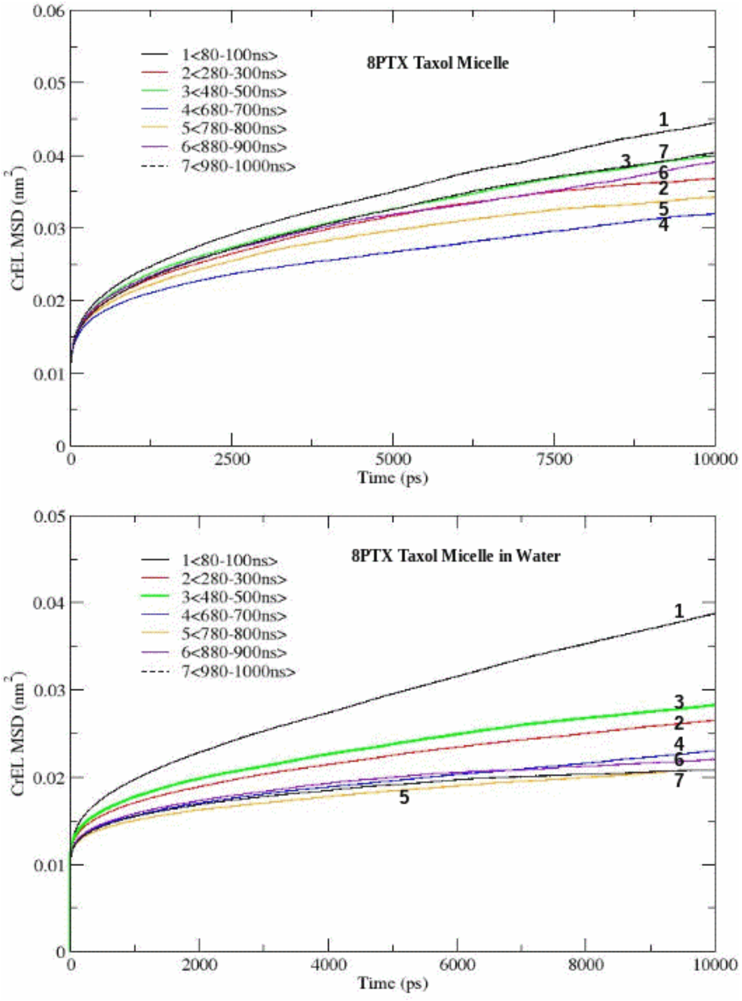

S5 Fig. CrEL mean square displacements in 8PTX Taxol micelle with and without water.

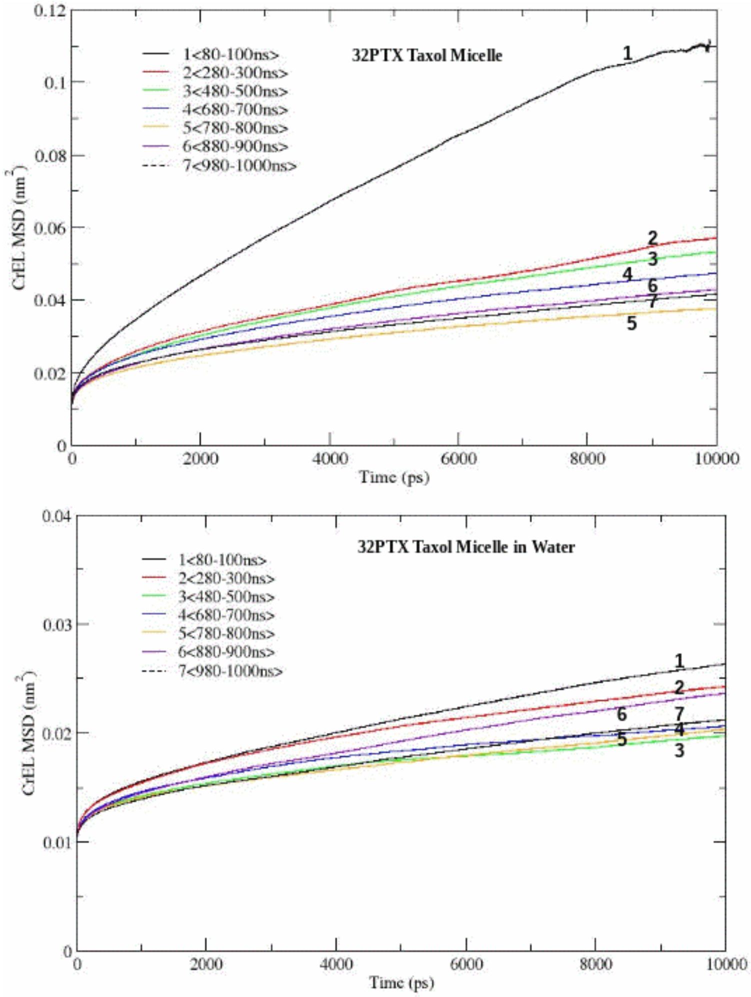

S6 Fig. CrEL mean square displacements in 32PTX Taxol micelle without and with water (average over last 20ns MD trajectories).

Radial Distribution Function

In order to better quantify the equilibrium trajectories, the radial distribution functions for the selected particle pairs are analyzed. Here, radial distribution function, $g_{AB}(r)$, between two particles of types A and B is defined as:

$g_{AB}(r) = \frac{\rho_B(r)}{\rho_B}$, where, $\rho_B(r)$ is the particle density of type B at a distance, r, around particles A, and $\rho_B(r)$ is the particle density of type B averaged over all spheres around particles A with radius, r_{max}. Here, the value of r_{max} is half of the box length. We use the analysis program g_rdf to calculate pair correlation functions.

In this analysis, RDFs were calculated for seven selected simulation trajectories of each 20ns run time. For the 3-wings CrEL molecule, we have selected 3 beads for the head (Gy1, Gy2, Gy3) and another 3 beads for the tail (CA4, CB4, CC4). For the PTX, the entire 24 beads molecule was chosen.

S7 Figure shows the radial distribution functions for the pairs (CA4-PTX) in the Taxol micelle without and with water cases. The without water case has the RDF plot shifted upward significantly over the 1000ns simulation. This is due to a compaction of the Taxol micelle. For the water case, there are no significant changes in the RDFs. In this case, the initial Taxol micelle in water was nearly in equilibrium and no significant relative mobility of the CrEL and PTX particles occurs. Here, CrEL and PTX are reorienting and the EOH particles are diffusing into water. The initial cubical micelle in water slowly transforms into a spherical shape.

S8 Figure shows the radial distribution functions for the pairs (Gy1-PTX) in the Taxol micelle without and with water. The local and bulk level changes are clearly noticeable at the earlier times. For case without water, the RDF plots are shifted upward significantly, and stabilized when the Taxol micelle is at steady state. For the water case, the RDFs shows several peaks and

no significant changes. This indicates that the spherical Taxol micelle in water is at equilibrium and well structured.

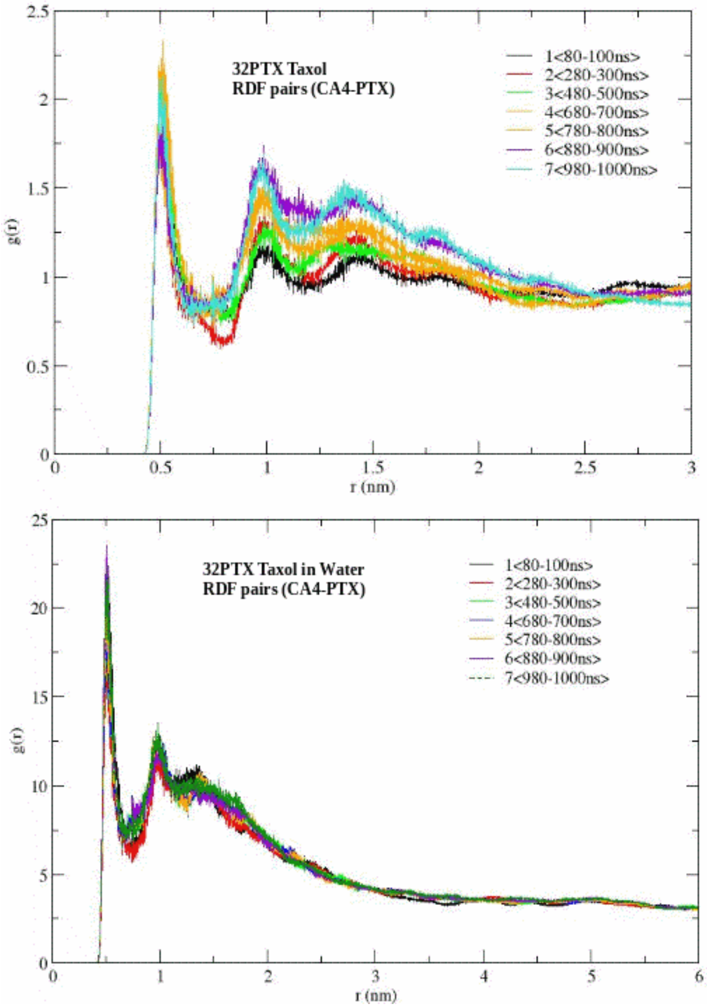

S7 Fig. Radial distribution functions for CrEL tail beads (CA4) and paclitaxel molecules (PTX) (average over last 20ns MD trajectories).

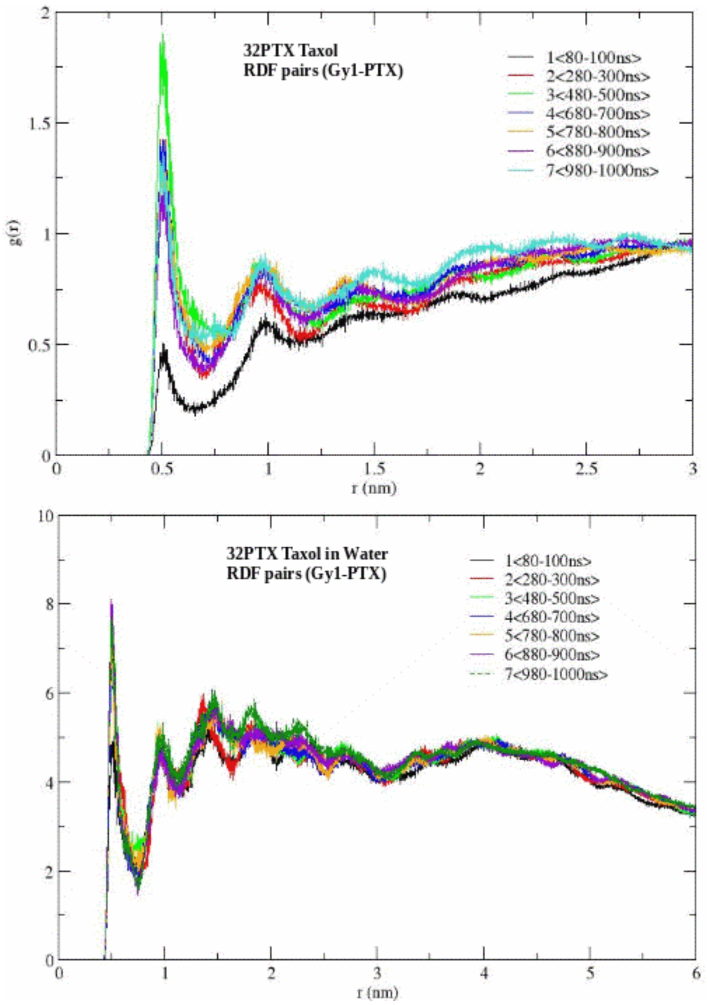

S8 Fig. Radial distribution functions for CrEL head beads (Gy1) and paclitaxel molecules (PTX) (average over last 20ns MD trajectories).

RMSD Analysis

To understand Taxol micelle evolution, we have calculated Pearson correlation and Spearman's rank correlation coefficients on RMSD data over the entire simulation period. Here we have added a sample of these analyses for clarification (S3 Table). These correlation coefficients showed a significant large positive relationship.

S3 Table. Pearson and Spearman Correlation Coefficients.

RMSD *(nm)* vs Times *(ns)* **(X-series vs Y-series)**	Pearson Correlation Coefficients		Spearman's Rank Correlation Coefficients	
	r_p (CrEL)	r_p (PTX)	r_s (CrEL)	r_s (PTX)
800-860	0.920	0.807	0.894	0.790
860-900	0.955	0.820	0.906	0.798
900-960	0.972	0.840	0.971	0.823
960-1000	0.973	0.834	0.970	0.770
