# Supplementary material for: Drug delivery process simulation—Quantifying the conformation dynamics of paclitaxel and cremophor EL
Source: PLoS One. 2025 May 12;20(5):e0313813. doi: 10.1371/journal.pone.0313813 (PMC12068633; doi:10.1371/journal.pone.0313813)
Supplement: S2 File — Molecular conformation analysis (S2_File.pdf): details of PTX and CrEL molecular conformations are analyzed for various binary PTX and CrEL systems, as well as ternary Taxol micelle cases, comparing both AA and CG molecular conformations. (PDF) [file pone.0313813.s002.pdf]

S2 File. Molecular Conformation

Drug Delivery Process Simulation - Quantifying the Conformation Dynamics of Paclitaxel and Cremophor EL

Mafiz Uddin[1*] and Dennis Coombe[2]

[1] Alberta Computational Biochemistry Lab, Edmonton, AB, Canada
[2] Computer Modelling Group, Calgary, AB, Canada

* Corresponding author
E-mail: mafiz.uddin36@gmail.com

PTX and CrEL Molecular Conformation

PTX and CrEL molecular conformations were analyzed for various binary and ternary Taxol micelle cases. By using energy minimization, energies for each conformation were calculated. The following MD systems were used to analysis both AA and CG molecular conformations: 8 PTX conformations in PTX-EOH and PTX-H2O binary systems (S9 Fig, S4 and S5 Tables); 32 PTX conformations in Taxol micelle (PTX-CrEL-EOH); 50 CrEL conformations in CrEL-EOH and CrEL-H2O binary systems (S10 to S12 Tables); 200 CrEL molecular conformations in Taxol micelle (PTX-CrEL-EOH)

In our paper, the conformation analysis for 32 PTX and 200 CrEL in the Taxol micelle were presented. Here, the corresponding conformation analysis for 8 PTX and 50 CrEL in the binary systems are given. Due to the large volume of data, the detailed energy statistical analyses are not included (this can be obtained from the authors by request).

General conclusions: a detailed statistical analysis of the relative conformational energies of CrEL showed that the spiral shape of 3 wings CrEL molecule is the dominant conformation. The lowest CrEL energy state was observed when the wings were fully closed. The highest energy state occurred when the wings were fully spread.

A detailed statistical analysis for PTX conformational energies was conducted and provided an energy frequency histogram. Lakdawala et al. (2001) focused on a butterfly conformation (one of their 7 PTX conformations). We also found these 7 conformations within our relative energy frequency histogram.

Binary System - PTX Conformation Analysis

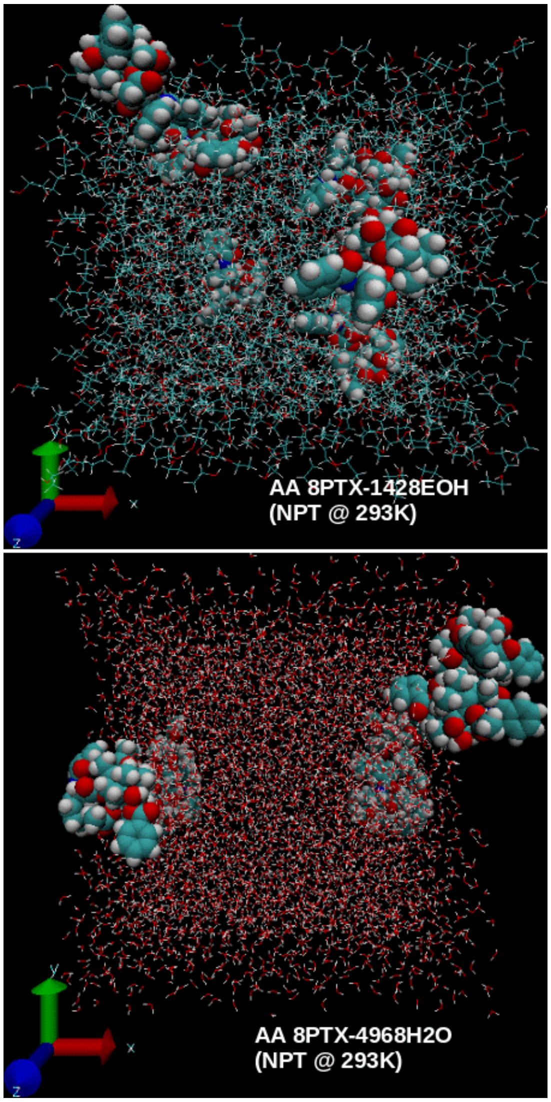

S9 Fig. AA MD atomic trajectories for the binary systems selected for PTX conformational analysis.

S4 Table. AA and CG PTX Conformational Energies in Ethanol.

# of PTX	AA MD				CG MD			
	$U\text{-}U^{ref}$ ($kJ.mol^{-1}$)	L_{12} (A)	L_{13} (A)	R_C (A)	$U\text{-}U^{ref}$ ($kJ.mol^{-1}$)	L_{12} (A)	L_{13} (A)	R_C (A)
1	6.890	12.712	10.368	5.05	52.250	16.634	10.692	7.742
2	15.127	12.668	8.316	5.263	0.000	13.101	5.392	6.82
3	0.000	11.313	9.569	4.923	43.140	18.249	10.663	7.545
4	8.014	11.229	6.758	5.056	39.743	9.872	13.67	7.642
5	16.847	12.099	8.048	5.056	51.848	15.397	12.939	6.942
6	17.124	10.992	8.256	5.123	61.087	14.335	15.28	7.183
7	18.189	11.92	8.532	5.023	51.744	15.293	16.505	7.6
8	6.671	12.038	7.662	5.259	3.546	8.754	5.336	6.873
Ave	11.108	11.871	8.439	5.094	37.920	13.954	11.310	7.293

U^{ref}=477.816 $kJ.mol^{-1}$ (AA), 21.762 $kJ.mol^{-1}$ (CG)

S5 Table. AA and CG PTX conformational energies in water.

# of PTX	AA MD				CG MD			
	$U\text{-}U^{ref}$ ($kJ.mol^{-1}$)	L_{12} (A)	L_{13} (A)	R_C(A)	$U\text{-}U^{ref}$ ($kJ.mol^{-1}$)	L_{12} (A)	L_{13} (A)	R_C (A)
1	27.098	11.395	7.440	4.831	12.231	4.990	10.179	6.956
2	0.000	11.730	6.496	5.066	41.526	5.025	13.021	7.267
3	14.504	12.185	7.899	5.132	0.000	4.746	5.243	5.714
4	41.936	11.453	9.482	4.997	53.186	17.139	16.558	8.226
5	9.976	11.196	9.076	4.890	56.086	9.560	13.594	6.745
6	31.009	11.932	9.018	5.104	32.195	6.497	9.621	6.924
7	19.348	12.154	7.092	5.089	48.457	14.663	13.280	6.966
8	47.622	10.599	8.358	5.350	26.932	5.964	15.162	6.900
Ave	23.937	11.581	8.108	5.057	28.573	8.573	12.082	6.962

U^{ref}=469.137 $kJ.mol^{-1}$ (AA), -15.000 $kJ.mol^{-1}$ (CG)

Binary System - CrEL Conformation Analysis

The three wings of the CrEL molecule have very complex conformational properties. The central polar segments have twisted conformations and the nonpolar tail segments have strong clustering properties. We first developed a correlation between potential energy and conformation (how CrEL potential energy changes with CrEL conformation). We have selected two MD cases of binary mixtures for this development: one case with 50 CrEL in ethanol and another case 50 CrEL in water (S10 Fig). At several simulation times (20, 40 and 100ns), the potential energy and atomic coordinates trajectory for each CrEL molecule were recorded. The frequency histogram of the 50 CrEL molecules was then plotted by dividing the entire energy spectrum into several energy groups.

Using OPLSA and MARTINI force fields, the conformational energies of 50 CrEL molecules in ethanol and water were calculated. In our paper, the computational algorithm calculating the radius of gyration (conformation) was given. The frequency histograms of the conformation energies for 50 CrEL molecules in ethanol and water were plotted in S11 Fig for AA and in S12 Fig for CG. These frequency plots can be described as left skewed Gaussian distributions. It should be noted that, in comparison with AA, the CG conformation energies were shifted significantly to lower energy states.

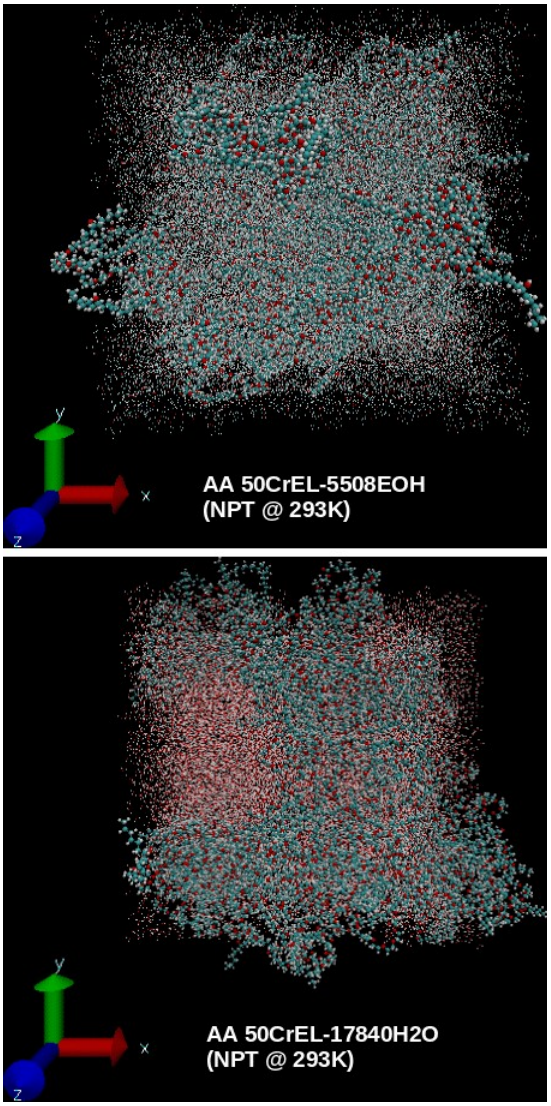

S10 Fig. AA MD atomic trajectories for the binary systems selected for CrEL conformational analysis.

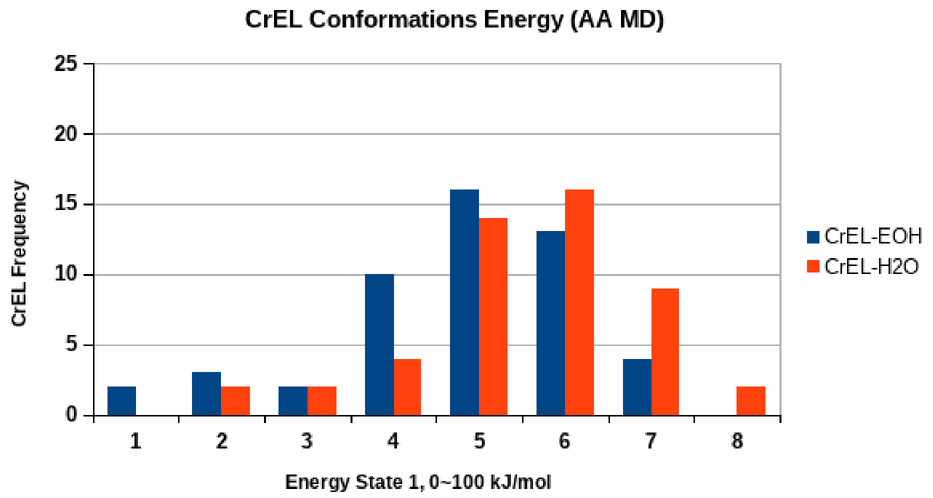

S11 Fig. Distribution of CrEL conformational energies for AA MD cases (CrEL-EOH) and (CrEL-H2O).

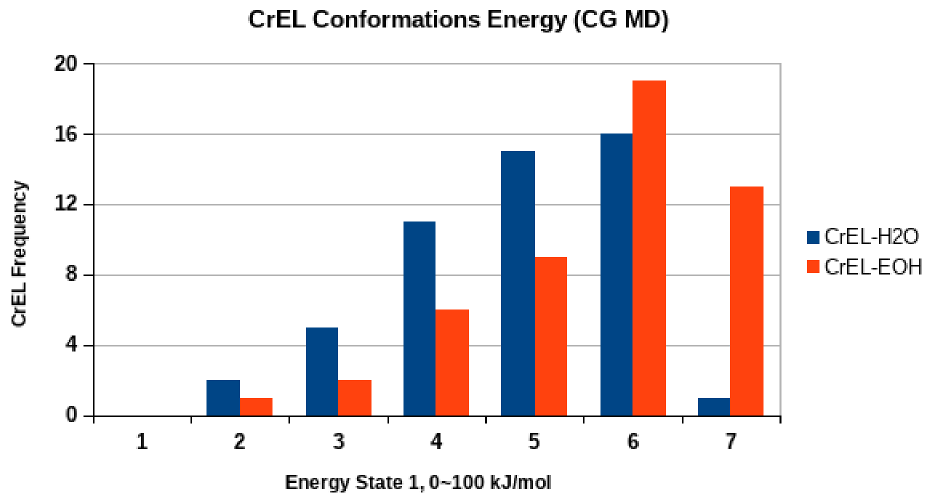

S12 Fig. Distribution of CrEL conformational energies for CG MD cases (CrEL-H2O) and (CrEL-EOH).
