# Supplementary material for: Drug delivery process simulation—Quantifying the conformation dynamics of paclitaxel and cremophor EL
Source: PLoS One. 2025 May 12;20(5):e0313813. doi: 10.1371/journal.pone.0313813 (PMC12068633; doi:10.1371/journal.pone.0313813)
Supplement: S3 File — PTX data(S3_File.pdf): CG and AA data for PTX molecules were included as supporting materials. (PDF) [file pone.0313813.s003.pdf]

S3 File. PTX Data

Drug Delivery Process Simulation - Quantifying the Conformation Dynamics of Paclitaxel and Cremophor EL

Mafiz Uddin[1*] and Dennis Coombe[2]

[1] Alberta Computational Biochemistry Lab, Edmonton, AB, Canada
[2] Computer Modelling Group, Calgary, AB, Canada

* Corresponding author
E-mail: mafiz.uddin36@gmail.com

; CG beads for molecule PTX (drug delivery research)

```
[ atoms ]
; id  type  resnr residu      atom  cgnr  charge      mass
  1   SC1   1     PTX         CR1   1     0.000       27.046
  2   C1    1     PTX         CA1   2     0.000       27.046
  3   SC1   1     PTX         CR2   3     0.000       25.030
  4   N0    1     PTX         ES1   4     0.000       59.0448
  5   C1    1     PTX         CA2   5     0.000       42.081
  6   P2    1     PTX         OH1   6     0.000       42.0374
  7   N0    1     PTX         ES2   7     0.000       44.0098
  8   SC5   1     PTX         R1    8     0.000       25.030
  9   SC5   1     PTX         R1    9     0.000       26.0348
 10   SC5   1     PTX         R1    10    0.000       26.038
 11   N0    1     PTX         ES3   11    0.000       44.0098
 12   P2    1     PTX         OH2   12    0.000       43.0454
 13   P3    1     PTX         NH    13    0.000       43.0251
 14   SC5   1     PTX         R2    14    0.000       25.030
 15   SC5   1     PTX         R2    15    0.000       26.038
 16   SC5   1     PTX         R2    16    0.000       26.038
 17   SC5   1     PTX         R3    17    0.000       25.030
 18   SC5   1     PTX         R3    18    0.000       26.038
 19   SC5   1     PTX         R3    19    0.000       26.038
 20   Na    1     PTX         KT    20    0.000       55.0564
 21   SC1   1     PTX         CR3   21    0.000       25.030
 22   N0    1     PTX         ES4   22    0.000       59.0448
 23   P2    1     PTX         OH3   23    0.000       44.0534
 24   N0    1     PTX         ET    24    0.000       43.0454
```

; CG bead coordinates for molecule PTX (drug delivery research)

```
; ptx_CG.gro (GROMACS), fixed format(i5,2a5,i5,3f8.3,3f8.4)
;rNum rName aName aNum  X        Y        Z
    1PTX    CR1    1   4.291   4.616   4.501
    1PTX    CA1    2   4.120   4.744   4.441
    1PTX    CR2    3   4.052   4.858   4.172
    1PTX    ES1    4   3.754   4.943   4.192
    1PTX    CA2    5   4.343   4.988   4.061
    1PTX    OH1    6   4.516   4.780   4.158
    1PTX    ES2    7   4.716   4.738   4.276
    1PTX     R1    8   4.902   4.791   4.486
    1PTX     R1    9   5.012   4.692   4.669
    1PTX     R1   10   4.862   4.810   4.741
    1PTX    ES3   11   4.329   4.391   4.811
    1PTX    OH2   12   4.357   4.230   4.994
    1PTX     NH   13   4.538   3.948   4.798
    1PTX     R2   14   4.618   3.822   4.545
    1PTX     R2   15   4.718   3.733   4.377
    1PTX     R2   16   4.639   3.832   4.329
    1PTX     R3   17   4.414   4.126   5.270
    1PTX     R3   18   4.560   4.116   5.451
    1PTX     R3   19   4.379   4.124   5.501
    1PTX     KT   20   4.086   4.603   3.961
    1PTX    CR3   21   4.466   4.548   3.872
    1PTX    ES4   22   4.800   4.544   3.796
    1PTX    OH3   23   4.010   4.504   3.768
    1PTX     ET   24   4.358   4.368   3.646
```

```
; AA directive for molecule PTX (drug delivery research)

[ atoms ]
;   nr       type  resnr residue  atom   cgnr     charge       mass
     1   opls_159    601    TXL     C1      1          0     12.011   ; qtot 0
     2   opls_154    601    TXL     O1      2       -0.5    15.9994   ; qtot -0.5
     3   opls_155    601    TXL     H1      2        0.5      1.008   ; qtot 0
     4   opls_137    601    TXL     C2      3      -0.14     12.011   ; qtot -0.14
     5   opls_140    601    TXL     H2      3       0.14      1.008   ; qtot 0
     6   opls_467    601    TXL     O2      4          0    15.9994   ; qtot 0
     7   opls_235    601    TXL     C3      4          0     12.011   ; qtot 0
     8   opls_236    601    TXL     O3      4          0    15.9994   ; qtot 0
     9   opls_145    601    TXL     C4      5          0     12.011   ; qtot 0
    10   opls_145    601    TXL     C5      6      -0.14     12.011   ; qtot -0.14
    11   opls_146    601    TXL     H5      6       0.14      1.008   ; qtot 0
    12   opls_145    601    TXL     C6      7      -0.14     12.011   ; qtot -0.14
    13   opls_146    601    TXL     H6      7       0.14      1.008   ; qtot 0
    14   opls_145    601    TXL     C7      8      -0.14     12.011   ; qtot -0.14
    15   opls_146    601    TXL     H7      8       0.14      1.008   ; qtot 0
    16   opls_145    601    TXL     C8      9      -0.14     12.011   ; qtot -0.14
    17   opls_146    601    TXL     H8      9       0.14      1.008   ; qtot 0
    18   opls_145    601    TXL     C9     10      -0.14     12.011   ; qtot -0.14
    19   opls_146    601    TXL     H9     10       0.14      1.008   ; qtot 0
    20   opls_137    601    TXL    C10     11      -0.14     12.011   ; qtot -0.14
    21   opls_140    601    TXL    H10     11       0.14      1.008   ; qtot 0
    22   opls_139    601    TXL    C11     12          0     12.011   ; qtot 0
    23   opls_467    601    TXL     O4     13          0    15.9994   ; qtot 0
    24   opls_465    601    TXL    C12     13          0     12.011   ; qtot 0
    25   opls_466    601    TXL     O5     13          0    15.9994   ; qtot 0
    26   opls_468    601    TXL    C13     14      -0.27     12.011   ; qtot -0.27
    27   opls_469    601    TXL   H131     14       0.09      1.008   ; qtot -0.18
    28   opls_469    601    TXL   H132     14       0.09      1.008   ; qtot -0.09
    29   opls_469    601    TXL   H133     14       0.09      1.008   ; qtot 0
    30   opls_182    601    TXL    C14     15      -0.14     12.011   ; qtot -0.14
    31   opls_185    601    TXL   H141     15       0.07      1.008   ; qtot -0.07
    32   opls_185    601    TXL   H142     15       0.07      1.008   ; qtot 0
    33   opls_186    601    TXL     O6     16          0    15.9994   ; qtot 0
    34   opls_183    601    TXL    C15     17      -0.14     12.011   ; qtot -0.14
    35   opls_185    601    TXL    H15     17       0.14      1.008   ; qtot 0
    36   opls_136    601    TXL    C16     18      -0.14     12.011   ; qtot -0.14
    37   opls_140    601    TXL   H161     18       0.07      1.008   ; qtot -0.07
    38   opls_140    601    TXL   H162     18       0.07      1.008   ; qtot 0
    39   opls_158    601    TXL    C17     19      -0.14     12.011   ; qtot -0.14
    40   opls_156    601    TXL    H17     19       0.14      1.008   ; qtot 0
    41   opls_154    601    TXL    O07     20       -0.4    15.9994   ; qtot -0.4
    42   opls_155    601    TXL    H07     20        0.4      1.008   ; qtot 0
    43   opls_139    601    TXL    C18     21          0     12.011   ; qtot 0
    44   opls_280    601    TXL    C20     22          0     12.011   ; qtot 0
    45   opls_281    601    TXL     O8     22          0    15.9994   ; qtot 0
    46   opls_137    601    TXL    C21     23      -0.14     12.011   ; qtot -0.14
    47   opls_140    601    TXL    H21     23       0.14      1.008   ; qtot 0
    48   opls_135    601    TXL    C19     24      -0.27     12.011   ; qtot -0.27
    49   opls_140    601    TXL   H191     24       0.09      1.008   ; qtot -0.18
    50   opls_140    601    TXL   H192     24       0.09      1.008   ; qtot -0.09
    51   opls_140    601    TXL   H193     24       0.09      1.008   ; qtot 0
```

52	opls_467	601	TXL	O9	25	0	15.9994	; qtot 0
53	opls_465	601	TXL	C22	25	0	12.011	; qtot 0
54	opls_466	601	TXL	O10	25	0	15.9994	; qtot 0
55	opls_468	601	TXL	C23	26	-0.27	12.011	; qtot -0.27
56	opls_469	601	TXL	H231	26	0.09	1.008	; qtot -0.18
57	opls_469	601	TXL	H232	26	0.09	1.008	; qtot -0.09
58	opls_469	601	TXL	H233	26	0.09	1.008	; qtot 0
59	opls_139	601	TXL	C24	27	0	12.011	; qtot 0
60	opls_141	601	TXL	C25	27	0	12.011	; qtot 0
61	opls_468	601	TXL	C26	28	-0.14	12.011	; qtot -0.14
62	opls_469	601	TXL	H26	28	0.14	1.008	; qtot 0
63	opls_467	601	TXL	O11	29	0	15.9994	; qtot 0
64	opls_465	601	TXL	C27	29	0	12.011	; qtot 0
65	opls_466	601	TXL	O12	29	0	15.9994	; qtot 0
66	opls_468	601	TXL	C28	30	-0.14	12.011	; qtot -0.14
67	opls_469	601	TXL	H28	30	0.14	1.008	; qtot 0
68	opls_154	601	TXL	O13	31	-0.14	15.9994	; qtot -0.14
69	opls_155	601	TXL	H13	31	0.14	1.008	; qtot 0
70	opls_137	601	TXL	C29	32	-0.14	12.011	; qtot -0.14
71	opls_140	601	TXL	H29	32	0.14	1.008	; qtot 0
72	opls_237	601	TXL	N01	33	-0.31	14.0067	; qtot -0.31
73	opls_240	601	TXL	H01	33	0.31	1.008	; qtot 0
74	opls_235	601	TXL	C30	34	0	12.011	; qtot 0
75	opls_236	601	TXL	O14	34	0	15.9994	; qtot 0
76	opls_145	601	TXL	C31	35	0	12.011	; qtot 0
77	opls_145	601	TXL	C32	36	-0.14	12.011	; qtot -0.14
78	opls_146	601	TXL	H32	36	0.14	1.008	; qtot 0
79	opls_145	601	TXL	C33	37	-0.14	12.011	; qtot -0.14
80	opls_146	601	TXL	H33	37	0.14	1.008	; qtot 0
81	opls_145	601	TXL	C34	38	-0.14	12.011	; qtot -0.14
82	opls_146	601	TXL	H34	38	0.14	1.008	; qtot 0
83	opls_145	601	TXL	C35	39	-0.14	12.011	; qtot -0.14
84	opls_146	601	TXL	H35	39	0.14	1.008	; qtot 0
85	opls_145	601	TXL	C36	40	-0.14	12.011	; qtot -0.14
86	opls_146	601	TXL	H36	40	0.14	1.008	; qtot 0
87	opls_232	601	TXL	C37	41	0	12.011	; qtot 0
88	opls_145	601	TXL	C38	42	-0.14	12.011	; qtot -0.14
89	opls_146	601	TXL	H38	42	0.14	1.008	; qtot 0
90	opls_145	601	TXL	C39	43	-0.14	12.011	; qtot -0.14
91	opls_146	601	TXL	H39	43	0.14	1.008	; qtot 0
92	opls_145	601	TXL	C40	44	-0.14	12.011	; qtot -0.14
93	opls_146	601	TXL	H40	44	0.14	1.008	; qtot 0
94	opls_145	601	TXL	C41	45	-0.14	12.011	; qtot -0.14
95	opls_146	601	TXL	H41	45	0.14	1.008	; qtot 0
96	opls_145	601	TXL	C42	46	-0.14	12.011	; qtot -0.14
97	opls_146	601	TXL	H42	46	0.14	1.008	; qtot 0
98	opls_136	601	TXL	C43	47	-0.14	12.011	; qtot -0.14
99	opls_140	601	TXL	H431	47	0.07	1.008	; qtot -0.07
100	opls_140	601	TXL	H432	47	0.07	1.008	; qtot 0
101	opls_135	601	TXL	C44	48	-0.27	12.011	; qtot -0.27
102	opls_140	601	TXL	H441	48	0.09	1.008	; qtot -0.18
103	opls_140	601	TXL	H442	48	0.09	1.008	; qtot -0.09
104	opls_140	601	TXL	H443	48	0.09	1.008	; qtot 0
105	opls_139	601	TXL	C45	49	0	12.011	; qtot 0
106	opls_135	601	TXL	C46	50	-0.27	12.011	; qtot -0.27
107	opls_140	601	TXL	H461	50	0.09	1.008	; qtot -0.18
108	opls_140	601	TXL	H462	50	0.09	1.008	; qtot -0.09

```
109   opls_140    601    TXL   H463     50        0.09      1.008   ; qtot 0
110   opls_135    601    TXL    C47     51       -0.27     12.011   ; qtot -0.27
111   opls_140    601    TXL   H471     51        0.09      1.008   ; qtot -0.18
112   opls_140    601    TXL   H472     51        0.09      1.008   ; qtot -0.09
113   opls_140    601    TXL   H473     51        0.09      1.008   ; qtot 0
```

; AA coordinates for molecule PTX (drug delivery research)

```
; ptx_aa.gro (GROMACS), fixed format(i5,2a5,i5,3f8.3,3f8.4)
;rNum rName aName aNum  X        Y        Z
    1TXL     C1    1   4.304   4.734   4.575
    1TXL     O1    2   4.184   4.779   4.637
    1TXL     H1    3   4.205   4.833   4.711
    1TXL     C2    4   4.389   4.669   4.691
    1TXL     H2    5   4.428   4.745   4.757
    1TXL     O2    6   4.296   4.591   4.766
    1TXL     C3    7   4.254   4.621   4.891
    1TXL     O3    8   4.295   4.713   4.962
    1TXL     C4    9   4.137   4.535   4.928
    1TXL     C5   10   4.070   4.459   4.829
    1TXL     H5   11   4.103   4.460   4.726
    1TXL     C6   12   3.956   4.384   4.862
    1TXL     H6   13   3.904   4.330   4.784
    1TXL     C7   14   3.910   4.381   4.994
    1TXL     H7   15   3.822   4.323   5.020
    1TXL     C8   16   3.977   4.455   5.094
    1TXL     H8   17   3.940   4.454   5.195
    1TXL     C9   18   4.090   4.532   5.061
    1TXL     H9   19   4.141   4.588   5.138
    1TXL    C10   20   4.510   4.576   4.650
    1TXL    H10   21   4.493   4.559   4.545
    1TXL    C11   22   4.501   4.431   4.708
    1TXL     O4   23   4.393   4.373   4.634
    1TXL    C12   24   4.332   4.257   4.659
    1TXL     O5   25   4.359   4.174   4.745
    1TXL    C13   26   4.221   4.237   4.558
    1TXL   H131   27   4.132   4.197   4.606
    1TXL   H132   28   4.195   4.333   4.514
    1TXL   H133   29   4.254   4.172   4.478
    1TXL    C14   30   4.499   4.415   4.860
    1TXL   H141   31   4.411   4.367   4.902
    1TXL   H142   32   4.521   4.507   4.915
    1TXL     O6   33   4.612   4.329   4.847
    1TXL    C15   34   4.625   4.341   4.705
    1TXL    H15   35   4.608   4.243   4.659
    1TXL    C16   36   4.760   4.402   4.670
    1TXL   H161   37   4.815   4.331   4.609
    1TXL   H162   38   4.819   4.414   4.761
    1TXL    C17   39   4.751   4.535   4.593
    1TXL    H17   40   4.719   4.509   4.492
    1TXL    O07   41   4.882   4.585   4.586
    1TXL    H07   42   4.910   4.607   4.674
    1TXL    C18   43   4.653   4.639   4.655
    1TXL    C20   44   4.659   4.768   4.569
    1TXL     O8   45   4.724   4.866   4.607
    1TXL    C21   46   4.609   4.763   4.425
    1TXL    H21   47   4.607   4.658   4.394
    1TXL    C19   48   4.697   4.675   4.800
    1TXL   H191   49   4.623   4.736   4.851
    1TXL   H192   50   4.713   4.587   4.861
    1TXL   H193   51   4.790   4.730   4.802
    1TXL     O9   52   4.703   4.835   4.346
```

1TXL	C22	53	4.754	4.785	4.232
1TXL	O10	54	4.730	4.674	4.183
1TXL	C23	55	4.859	4.877	4.172
1TXL	H231	56	4.945	4.883	4.239
1TXL	H232	57	4.892	4.836	4.076
1TXL	H233	58	4.820	4.977	4.156
1TXL	C24	59	4.468	4.821	4.398
1TXL	C25	60	4.396	4.738	4.294
1TXL	C26	61	4.324	4.612	4.335
1TXL	H26	62	4.245	4.600	4.261
1TXL	O11	63	4.397	4.490	4.336
1TXL	C27	64	4.385	4.397	4.239
1TXL	O12	65	4.303	4.397	4.147
1TXL	C28	66	4.497	4.295	4.249
1TXL	H28	67	4.520	4.293	4.356
1TXL	O13	68	4.611	4.348	4.183
1TXL	H13	69	4.604	4.329	4.091
1TXL	C29	70	4.463	4.149	4.205
1TXL	H29	71	4.356	4.134	4.219
1TXL	N01	72	4.503	4.121	4.066
1TXL	H01	73	4.600	4.099	4.050
1TXL	C30	74	4.415	4.108	3.965
1TXL	O14	75	4.295	4.127	3.980
1TXL	C31	76	4.461	4.034	3.842
1TXL	C32	77	4.576	4.075	3.771
1TXL	H32	78	4.638	4.155	3.808
1TXL	C33	79	4.606	4.018	3.645
1TXL	H33	80	4.690	4.054	3.588
1TXL	C34	81	4.524	3.915	3.593
1TXL	H34	82	4.547	3.872	3.497
1TXL	C35	83	4.414	3.869	3.667
1TXL	H35	84	4.352	3.789	3.628
1TXL	C36	85	4.382	3.929	3.790
1TXL	H36	86	4.295	3.896	3.845
1TXL	C37	87	4.530	4.046	4.296
1TXL	C38	88	4.668	4.078	4.345
1TXL	H38	89	4.732	4.142	4.286
1TXL	C39	90	4.709	4.032	4.471
1TXL	H39	91	4.808	4.059	4.508
1TXL	C40	92	4.624	3.954	4.552
1TXL	H40	93	4.660	3.919	4.648
1TXL	C41	94	4.493	3.923	4.512
1TXL	H41	95	4.429	3.866	4.578
1TXL	C42	96	4.442	3.968	4.388
1TXL	H42	97	4.340	3.945	4.361
1TXL	C43	98	4.247	4.634	4.468
1TXL	H431	99	4.152	4.674	4.433
1TXL	H432	100	4.218	4.539	4.513
1TXL	C44	101	4.397	4.782	4.150
1TXL	H441	102	4.358	4.884	4.143
1TXL	H442	103	4.498	4.781	4.111
1TXL	H443	104	4.335	4.716	4.088
1TXL	C45	105	4.366	4.862	4.510
1TXL	C46	106	4.256	4.944	4.437
1TXL	H461	107	4.187	4.992	4.505
1TXL	H462	108	4.302	5.022	4.375
1TXL	H463	109	4.196	4.883	4.370

1TXL	C47	110	4.422	4.966	4.612
1TXL	H471	111	4.478	5.042	4.558
1TXL	H472	112	4.342	5.016	4.666
1TXL	H473	113	4.489	4.929	4.688
