# Supplementary material for: Drug delivery process simulation—Quantifying the conformation dynamics of paclitaxel and cremophor EL
Source: PLoS One. 2025 May 12;20(5):e0313813. doi: 10.1371/journal.pone.0313813 (PMC12068633; doi:10.1371/journal.pone.0313813)
Supplement: S4 File — CrEL data (S4_File.pdf): CG and AA data for CrEL molecules were included as supporting materials. (PDF) [file pone.0313813.s004.pdf]

S4 File. CrEL Data

Drug Delivery Process Simulation - Quantifying the Conformation Dynamics of Paclitaxel and Cremophor EL

Mafiz Uddin[1*] and Dennis Coombe[2]

[1] Alberta Computational Biochemistry Lab, Edmonton, AB, Canada
[2] Computer Modelling Group, Calgary, AB, Canada

* Corresponding author
E-mail: mafiz.uddin36@gmail.com

; CG beads for molecule CREL (drug delivery research)

```
[ atoms ]
; id  type  resnr      residu      atom  cgnr  charge   mass
   1    P1        1    CREL     Gy1        1       0.000   58.0804
   2    P2        1    CREL     EO1        2       0.000   44.0534
   3    P2        1    CREL     EO2        3       0.000   44.0534
   4    P2        1    CREL     EO3        4       0.000   44.0534
   5    P2        1    CREL     EO4        5       0.000   44.0534
   6    P2        1    CREL     EO5        6       0.000   44.0534
   7    P2        1    CREL     EO6        7       0.000   44.0534
   8    P2        1    CREL     EO7        8       0.000   44.0534
   9    P2        1    CREL     EO8        9       0.000   44.0534
  10    P2        1    CREL     EO9       10       0.000   44.0534
  11    P2        1    CREL     EO10      11       0.000   44.0534
  12    P2        1    CREL     EO11      12       0.000   44.0534
  13    P3        1    CREL     COO1      13       0.000   58.0368
  14    C1        1    CREL     CA1       14       0.000   56.1080
  15    C3        1    CREL     CD1       15       0.000   26.0380
  16    C1        1    CREL     CA2       16       0.000   41.0730
  17    SP2       1    CREL     OH1       17       0.000   17.0074
  18    C1        1    CREL     CA3       18       0.000   42.0810
  19    C1        1    CREL     CA4       19       0.000   43.0890
  20    P1        1    CREL     Gy2       20       0.000   57.0724
  21    P2        1    CREL     EO12      21       0.000   44.0534
  22    P2        1    CREL     EO13      22       0.000   44.0534
  23    P2        1    CREL     EO14      23       0.000   44.0534
  24    P2        1    CREL     EO15      24       0.000   44.0534
  25    P2        1    CREL     EO16      25       0.000   44.0534
  26    P2        1    CREL     EO17      26       0.000   44.0534
  27    P2        1    CREL     EO18      27       0.000   44.0534
  28    P2        1    CREL     EO19      28       0.000   44.0534
  29    P2        1    CREL     EO20      29       0.000   44.0534
  30    P2        1    CREL     EO21      30       0.000   44.0534
  31    P2        1    CREL     EO22      31       0.000   44.0534
  32    P3        1    CREL     COO2      32       0.000   58.0368
  33    C1        1    CREL     CB1       33       0.000   56.1080
  34    C3        1    CREL     CD2       34       0.000   26.0380
  35    C1        1    CREL     CB2       35       0.000   41.0730
  36    SP2       1    CREL     OH2       36       0.000   17.0074
  37    C1        1    CREL     CB3       37       0.000   42.0810
  38    C1        1    CREL     CB4       38       0.000   43.0890
  39    P1        1    CREL     Gy3       39       0.000   58.0804
  40    P2        1    CREL     EO23      40       0.000   44.0534
  41    P2        1    CREL     EO24      41       0.000   44.0534
  42    P2        1    CREL     EO25      42       0.000   44.0534
  43    P2        1    CREL     EO26      43       0.000   44.0534
  44    P2        1    CREL     EO27      44       0.000   44.0534
  45    P2        1    CREL     EO28      45       0.000   44.0534
  46    P2        1    CREL     EO29      46       0.000   44.0534
  47    P2        1    CREL     EO30      47       0.000   44.0534
  48    P2        1    CREL     EO31      48       0.000   44.0534
  49    P2        1    CREL     EO32      49       0.000   44.0534
  50    P2        1    CREL     EO33      50       0.000   44.0534
```

51	P3	1	CREL	COO3	51	0.000	58.0368
52	C1	1	CREL	CC1	52	0.000	56.1080
53	C3	1	CREL	CD3	53	0.000	26.0380
54	C1	1	CREL	CC2	54	0.000	41.0730
55	SP2	1	CREL	OH3	55	0.000	17.0074
56	C1	1	CREL	CC3	56	0.000	42.0810
57	C1	1	CREL	CC4	57	0.000	43.0890

; CG bead coordinates for molecule CREL (drug delivery research)

```
; crel_cg.gro (GROMACS), Fixed Format(i5,2a5,i5,3f8.3,3f8.4)
;rNum rName aName aNum  X        Y        Z
    1CREL   Gy1    1   2.873   4.790   4.702
    1CREL   EO1    2   3.044   4.532   4.482
    1CREL   EO2    3   3.140   4.254   4.342
    1CREL   EO3    4   3.259   3.996   4.179
    1CREL   EO4    5   3.395   3.736   4.036
    1CREL   EO5    6   3.542   3.449   4.007
    1CREL   EO6    7   3.755   3.206   4.058
    1CREL   EO7    8   3.993   2.983   4.094
    1CREL   EO8    9   4.298   2.861   4.065
    1CREL   EO9   10   4.617   2.847   4.150
    1CREL  EO10   11   4.929   2.909   4.229
    1CREL  EO11   12   5.176   3.105   4.267
    1CREL  COO1   13   5.334   3.339   4.142
    1CREL   CA1   14   5.181   3.702   3.968
    1CREL   CD1   15   4.931   3.895   3.893
    1CREL   CA2   16   4.605   3.964   4.036
    1CREL   OH1   17   4.511   3.718   4.024
    1CREL   CA3   18   4.237   4.125   4.167
    1CREL   CA4   19   3.962   4.355   4.308
    1CREL   Gy2   20   3.099   5.043   4.862
    1CREL  EO12   21   3.399   4.836   4.880
    1CREL  EO13   22   3.686   4.691   4.882
    1CREL  EO14   23   3.870   4.424   4.844
    1CREL  EO15   24   4.098   4.202   4.771
    1CREL  EO16   25   4.327   4.016   4.631
    1CREL  EO17   26   4.568   3.834   4.520
    1CREL  EO18   27   4.848   3.684   4.494
    1CREL  EO19   28   5.081   3.515   4.635
    1CREL  EO20   29   5.358   3.355   4.662
    1CREL  EO21   30   5.652   3.280   4.549
    1CREL  EO22   31   5.945   3.372   4.449
    1CREL  COO2   32   6.115   3.633   4.395
    1CREL   CB1   33   6.143   4.063   4.419
    1CREL   CD2   34   5.973   4.332   4.420
    1CREL   CB2   35   5.640   4.459   4.426
    1CREL   OH2   36   5.494   4.274   4.538
    1CREL   CB3   37   5.275   4.643   4.331
    1CREL   CB4   38   4.950   4.841   4.342
    1CREL   Gy3   39   3.094   5.179   5.163
    1CREL  EO23   40   2.908   5.492   5.085
    1CREL  EO24   41   2.790   5.724   4.894
    1CREL  EO25   42   2.822   5.829   4.593
    1CREL  EO26   43   2.913   5.717   4.306
    1CREL  EO27   44   3.036   5.546   4.069
    1CREL  EO28   45   3.273   5.371   3.968
    1CREL  EO29   46   3.561   5.278   4.023
    1CREL  EO30   47   3.855   5.213   4.092
    1CREL  EO31   48   4.113   5.240   4.266
    1CREL  EO32   49   4.338   5.334   4.459
    1CREL  EO33   50   4.574   5.487   4.578
    1CREL  COO3   51   4.810   5.659   4.659
    1CREL   CC1   52   5.234   5.625   4.728
```

1CREL	CD3	53	5.558	5.665	4.739
1CREL	CC2	54	5.899	5.732	4.832
1CREL	OH3	55	5.970	5.920	4.664
1CREL	CC3	56	6.228	5.775	5.084
1CREL	CC4	57	6.472	5.711	5.366

; AA directive for molecule CREL (drug delivery research)

```
[ atoms ]
;   nr       type  resnr residue  atom   cgnr     charge       mass
     1   opls_173      1   CREL    C01      1        0.2     12.011   ; qtot 0.2
     2   opls_176      1   CREL   H011      1       0.06      1.008   ; qtot 0.26
     3   opls_176      1   CREL   H012      1       0.06      1.008   ; qtot 0.32
     4   opls_171      1   CREL    O02      2       -0.3    15.9994   ; qtot 0.02
     5   opls_174      1   CREL    C03      3        0.2     12.011   ; qtot 0.22
     6   opls_176      1   CREL    H03      3       0.06      1.008   ; qtot 0.28
     7   opls_171      1   CREL    O04      4       -0.3    15.9994   ; qtot -0.02
     8   opls_173      1   CREL    C05      5        0.2     12.011   ; qtot 0.18
     9   opls_176      1   CREL   H051      5       0.06      1.008   ; qtot 0.24
    10   opls_176      1   CREL   H052      5       0.06      1.008   ; qtot 0.3
    11   opls_171      1   CREL    O06      6       -0.3    15.9994   ; qtot 0
    12   opls_182      1   CREL    C07      7       0.14     12.011   ; qtot 0.14
    13   opls_185      1   CREL   H071      7       0.03      1.008   ; qtot 0.17
    14   opls_185      1   CREL   H072      7       0.03      1.008   ; qtot 0.2
    15   opls_182      1   CREL    C08      8       0.14     12.011   ; qtot 0.34
    16   opls_185      1   CREL   H081      8       0.03      1.008   ; qtot 0.37
    17   opls_185      1   CREL   H082      8       0.03      1.008   ; qtot 0.4
    18   opls_180      1   CREL    O09      9       -0.4    15.9994   ; qtot 0
    19   opls_182      1   CREL    C10     10       0.14     12.011   ; qtot 0.14
    20   opls_185      1   CREL   H101     10       0.03      1.008   ; qtot 0.17
    21   opls_185      1   CREL   H102     10       0.03      1.008   ; qtot 0.2
    22   opls_182      1   CREL    C11     11       0.14     12.011   ; qtot 0.34
    23   opls_185      1   CREL   H111     11       0.03      1.008   ; qtot 0.37
    24   opls_185      1   CREL   H112     11       0.03      1.008   ; qtot 0.4
    25   opls_180      1   CREL    O12     12       -0.4    15.9994   ; qtot 0
    26   opls_182      1   CREL    C13     13       0.14     12.011   ; qtot 0.14
    27   opls_185      1   CREL   H131     13       0.03      1.008   ; qtot 0.17
    28   opls_185      1   CREL   H132     13       0.03      1.008   ; qtot 0.2
    29   opls_182      1   CREL    C14     14       0.14     12.011   ; qtot 0.34
    30   opls_185      1   CREL   H141     14       0.03      1.008   ; qtot 0.37
    31   opls_185      1   CREL   H142     14       0.03      1.008   ; qtot 0.4
    32   opls_180      1   CREL    O15     15       -0.4    15.9994   ; qtot 0
    33   opls_182      1   CREL    C16     16       0.14     12.011   ; qtot 0.14
    34   opls_185      1   CREL   H161     16       0.03      1.008   ; qtot 0.17
    35   opls_185      1   CREL   H162     16       0.03      1.008   ; qtot 0.2
    36   opls_182      1   CREL    C17     17       0.14     12.011   ; qtot 0.34
    37   opls_185      1   CREL   H171     17       0.03      1.008   ; qtot 0.37
    38   opls_185      1   CREL   H172     17       0.03      1.008   ; qtot 0.4
    39   opls_180      1   CREL    O18     18       -0.4    15.9994   ; qtot 0
    40   opls_182      1   CREL    C19     19       0.14     12.011   ; qtot 0.14
    41   opls_185      1   CREL   H191     19       0.03      1.008   ; qtot 0.17
    42   opls_185      1   CREL   H192     19       0.03      1.008   ; qtot 0.2
    43   opls_182      1   CREL    C20     20       0.14     12.011   ; qtot 0.34
    44   opls_185      1   CREL   H201     20       0.03      1.008   ; qtot 0.37
    45   opls_185      1   CREL   H202     20       0.03      1.008   ; qtot 0.4
    46   opls_180      1   CREL    O21     21       -0.4    15.9994   ; qtot 0
    47   opls_182      1   CREL    C22     22       0.14     12.011   ; qtot 0.14
    48   opls_185      1   CREL   H221     22       0.03      1.008   ; qtot 0.17
    49   opls_185      1   CREL   H222     22       0.03      1.008   ; qtot 0.2
    50   opls_182      1   CREL    C23     23       0.14     12.011   ; qtot 0.34
    51   opls_185      1   CREL   H231     23       0.03      1.008   ; qtot 0.37
```

52	opls_185	1	CREL	H232	23	0.03	1.008	; qtot 0.4
53	opls_180	1	CREL	O24	24	-0.4	15.9994	; qtot 0
54	opls_182	1	CREL	C25	25	0.14	12.011	; qtot 0.14
55	opls_185	1	CREL	H251	25	0.03	1.008	; qtot 0.17
56	opls_185	1	CREL	H252	25	0.03	1.008	; qtot 0.2
57	opls_182	1	CREL	C26	26	0.14	12.011	; qtot 0.34
58	opls_185	1	CREL	H261	26	0.03	1.008	; qtot 0.37
59	opls_185	1	CREL	H262	26	0.03	1.008	; qtot 0.4
60	opls_180	1	CREL	O27	27	-0.4	15.9994	; qtot 0
61	opls_182	1	CREL	C28	28	0.14	12.011	; qtot 0.14
62	opls_185	1	CREL	H281	28	0.03	1.008	; qtot 0.17
63	opls_185	1	CREL	H282	28	0.03	1.008	; qtot 0.2
64	opls_182	1	CREL	C29	29	0.14	12.011	; qtot 0.34
65	opls_185	1	CREL	H291	29	0.03	1.008	; qtot 0.37
66	opls_185	1	CREL	H292	29	0.03	1.008	; qtot 0.4
67	opls_180	1	CREL	O30	30	-0.4	15.9994	; qtot 0
68	opls_182	1	CREL	C31	31	0.14	12.011	; qtot 0.14
69	opls_185	1	CREL	H311	31	0.03	1.008	; qtot 0.17
70	opls_185	1	CREL	H312	31	0.03	1.008	; qtot 0.2
71	opls_182	1	CREL	C32	32	0.14	12.011	; qtot 0.34
72	opls_185	1	CREL	H321	32	0.03	1.008	; qtot 0.37
73	opls_185	1	CREL	H322	32	0.03	1.008	; qtot 0.4
74	opls_180	1	CREL	O33	33	-0.4	15.9994	; qtot 0
75	opls_182	1	CREL	C34	34	0.14	12.011	; qtot 0.14
76	opls_185	1	CREL	H341	34	0.03	1.008	; qtot 0.17
77	opls_185	1	CREL	H342	34	0.03	1.008	; qtot 0.2
78	opls_182	1	CREL	C35	35	0.14	12.011	; qtot 0.34
79	opls_185	1	CREL	H351	35	0.03	1.008	; qtot 0.37
80	opls_185	1	CREL	H352	35	0.03	1.008	; qtot 0.4
81	opls_180	1	CREL	O36	36	-0.4	15.9994	; qtot 0
82	opls_182	1	CREL	C37	37	0.14	12.011	; qtot 0.14
83	opls_185	1	CREL	H371	37	0.03	1.008	; qtot 0.17
84	opls_185	1	CREL	H372	37	0.03	1.008	; qtot 0.2
85	opls_182	1	CREL	C38	38	0.14	12.011	; qtot 0.34
86	opls_185	1	CREL	H381	38	0.03	1.008	; qtot 0.37
87	opls_185	1	CREL	H382	38	0.03	1.008	; qtot 0.4
88	opls_180	1	CREL	O39	39	-0.4	15.9994	; qtot 0
89	opls_182	1	CREL	C40	40	0.14	12.011	; qtot 0.14
90	opls_185	1	CREL	H401	40	0.03	1.008	; qtot 0.17
91	opls_185	1	CREL	H402	40	0.03	1.008	; qtot 0.2
92	opls_182	1	CREL	C41	41	0.22	12.011	; qtot 0.42
93	opls_185	1	CREL	H411	41	0.06	1.008	; qtot 0.48
94	opls_185	1	CREL	H412	41	0.06	1.008	; qtot 0.54
95	opls_278	1	CREL	O42	42	-0.44	15.9994	; qtot 0.1
96	opls_280	1	CREL	C43	43	0.47	12.011	; qtot 0.57
97	opls_281	1	CREL	O44	44	-0.47	15.9994	; qtot 0.1
98	opls_136	1	CREL	C45	45	-0.12	12.011	; qtot -0.02
99	opls_140	1	CREL	H451	45	0.06	1.008	; qtot 0.04
100	opls_140	1	CREL	H452	45	0.06	1.008	; qtot 0.1
101	opls_136	1	CREL	C46	46	-0.12	12.011	; qtot -0.02
102	opls_140	1	CREL	H461	46	0.06	1.008	; qtot 0.04
103	opls_140	1	CREL	H462	46	0.06	1.008	; qtot 0.1
104	opls_136	1	CREL	C47	47	-0.12	12.011	; qtot -0.02
105	opls_140	1	CREL	H471	47	0.06	1.008	; qtot 0.04
106	opls_140	1	CREL	H472	47	0.06	1.008	; qtot 0.1
107	opls_136	1	CREL	C48	48	-0.12	12.011	; qtot -0.02
108	opls_140	1	CREL	H481	48	0.06	1.008	; qtot 0.04

109	opls_140	1	CREL	H482	48	0.06	1.008	; qtot 0.1
110	opls_136	1	CREL	C49	49	-0.12	12.011	; qtot -0.02
111	opls_140	1	CREL	H491	49	0.06	1.008	; qtot 0.04
112	opls_140	1	CREL	H492	49	0.06	1.008	; qtot 0.1
113	opls_136	1	CREL	C50	50	-0.12	12.011	; qtot -0.02
114	opls_140	1	CREL	H501	50	0.06	1.008	; qtot 0.04
115	opls_140	1	CREL	H502	50	0.06	1.008	; qtot 0.1
116	opls_142	1	CREL	C51	51	-0.115	12.011	; qtot -0.015
117	opls_144	1	CREL	H51	51	0.115	1.008	; qtot 0.1
118	opls_142	1	CREL	C52	52	-0.115	12.011	; qtot -0.015
119	opls_144	1	CREL	H52	52	0.115	1.008	; qtot 0.1
120	opls_136	1	CREL	C53	53	-0.12	12.011	; qtot -0.02
121	opls_140	1	CREL	H531	53	0.06	1.008	; qtot 0.04
122	opls_140	1	CREL	H532	53	0.06	1.008	; qtot 0.1
123	opls_136	1	CREL	C54	54	-0.12	12.011	; qtot -0.02
124	opls_140	1	CREL	H541	54	0.06	1.008	; qtot 0.04
125	opls_140	1	CREL	H542	54	0.06	1.008	; qtot 0.1
126	opls_137	1	CREL	C55	55	-0.06	12.011	; qtot 0.04
127	opls_140	1	CREL	H55	55	0.06	1.008	; qtot 0.1
128	opls_434	1	CREL	O56	56	-0.6	15.9994	; qtot -0.5
129	opls_435	1	CREL	H56	56	0.6	1.008	; qtot 0.1
130	opls_136	1	CREL	C57	57	-0.12	12.011	; qtot -0.02
131	opls_140	1	CREL	H571	57	0.06	1.008	; qtot 0.04
132	opls_140	1	CREL	H572	57	0.06	1.008	; qtot 0.1
133	opls_136	1	CREL	C58	58	-0.12	12.011	; qtot -0.02
134	opls_140	1	CREL	H581	58	0.06	1.008	; qtot 0.04
135	opls_140	1	CREL	H582	58	0.06	1.008	; qtot 0.1
136	opls_136	1	CREL	C59	59	-0.12	12.011	; qtot -0.02
137	opls_140	1	CREL	H591	59	0.06	1.008	; qtot 0.04
138	opls_140	1	CREL	H592	59	0.06	1.008	; qtot 0.1
139	opls_136	1	CREL	C60	60	-0.12	12.011	; qtot -0.02
140	opls_140	1	CREL	H601	60	0.06	1.008	; qtot 0.04
141	opls_140	1	CREL	H602	60	0.06	1.008	; qtot 0.1
142	opls_136	1	CREL	C61	61	-0.12	12.011	; qtot -0.02
143	opls_140	1	CREL	H611	61	0.06	1.008	; qtot 0.04
144	opls_140	1	CREL	H612	61	0.06	1.008	; qtot 0.1
145	opls_135	1	CREL	C62	62	-0.18	12.011	; qtot -0.08
146	opls_140	1	CREL	H621	62	0.06	1.008	; qtot -0.02
147	opls_140	1	CREL	H622	62	0.06	1.008	; qtot 0.04
148	opls_140	1	CREL	H623	62	0.06	1.008	; qtot 0.1
149	opls_182	1	CREL	C63	63	0.14	12.011	; qtot 0.24
150	opls_185	1	CREL	H631	63	0.03	1.008	; qtot 0.27
151	opls_185	1	CREL	H632	63	0.03	1.008	; qtot 0.3
152	opls_182	1	CREL	C64	64	0.14	12.011	; qtot 0.44
153	opls_185	1	CREL	H641	64	0.03	1.008	; qtot 0.47
154	opls_185	1	CREL	H642	64	0.03	1.008	; qtot 0.5
155	opls_180	1	CREL	O65	65	-0.4	15.9994	; qtot 0.1
156	opls_182	1	CREL	C66	66	0.14	12.011	; qtot 0.24
157	opls_185	1	CREL	H661	66	0.03	1.008	; qtot 0.27
158	opls_185	1	CREL	H662	66	0.03	1.008	; qtot 0.3
159	opls_182	1	CREL	C67	67	0.14	12.011	; qtot 0.44
160	opls_185	1	CREL	H671	67	0.03	1.008	; qtot 0.47
161	opls_185	1	CREL	H672	67	0.03	1.008	; qtot 0.5
162	opls_180	1	CREL	O68	68	-0.4	15.9994	; qtot 0.1
163	opls_182	1	CREL	C69	69	0.14	12.011	; qtot 0.24
164	opls_185	1	CREL	H691	69	0.03	1.008	; qtot 0.27
165	opls_185	1	CREL	H692	69	0.03	1.008	; qtot 0.3

166	opls_182	1	CREL	C70	70	0.14	12.011	; qtot 0.44
167	opls_185	1	CREL	H701	70	0.03	1.008	; qtot 0.47
168	opls_185	1	CREL	H702	70	0.03	1.008	; qtot 0.5
169	opls_180	1	CREL	O71	71	-0.4	15.9994	; qtot 0.1
170	opls_182	1	CREL	C72	72	0.14	12.011	; qtot 0.24
171	opls_185	1	CREL	H721	72	0.03	1.008	; qtot 0.27
172	opls_185	1	CREL	H722	72	0.03	1.008	; qtot 0.3
173	opls_182	1	CREL	C73	73	0.14	12.011	; qtot 0.44
174	opls_185	1	CREL	H731	73	0.03	1.008	; qtot 0.47
175	opls_185	1	CREL	H732	73	0.03	1.008	; qtot 0.5
176	opls_180	1	CREL	O74	74	-0.4	15.9994	; qtot 0.1
177	opls_182	1	CREL	C75	75	0.14	12.011	; qtot 0.24
178	opls_185	1	CREL	H751	75	0.03	1.008	; qtot 0.27
179	opls_185	1	CREL	H752	75	0.03	1.008	; qtot 0.3
180	opls_182	1	CREL	C76	76	0.14	12.011	; qtot 0.44
181	opls_185	1	CREL	H761	76	0.03	1.008	; qtot 0.47
182	opls_185	1	CREL	H762	76	0.03	1.008	; qtot 0.5
183	opls_180	1	CREL	O77	77	-0.4	15.9994	; qtot 0.1
184	opls_182	1	CREL	C78	78	0.14	12.011	; qtot 0.24
185	opls_185	1	CREL	H781	78	0.03	1.008	; qtot 0.27
186	opls_185	1	CREL	H782	78	0.03	1.008	; qtot 0.3
187	opls_182	1	CREL	C79	79	0.14	12.011	; qtot 0.44
188	opls_185	1	CREL	H791	79	0.03	1.008	; qtot 0.47
189	opls_185	1	CREL	H792	79	0.03	1.008	; qtot 0.5
190	opls_180	1	CREL	O80	80	-0.4	15.9994	; qtot 0.1
191	opls_182	1	CREL	C81	81	0.14	12.011	; qtot 0.24
192	opls_185	1	CREL	H811	81	0.03	1.008	; qtot 0.27
193	opls_185	1	CREL	H812	81	0.03	1.008	; qtot 0.3
194	opls_182	1	CREL	C82	82	0.14	12.011	; qtot 0.44
195	opls_185	1	CREL	H821	82	0.03	1.008	; qtot 0.47
196	opls_185	1	CREL	H822	82	0.03	1.008	; qtot 0.5
197	opls_180	1	CREL	O83	83	-0.4	15.9994	; qtot 0.1
198	opls_182	1	CREL	C84	84	0.14	12.011	; qtot 0.24
199	opls_185	1	CREL	H841	84	0.03	1.008	; qtot 0.27
200	opls_185	1	CREL	H842	84	0.03	1.008	; qtot 0.3
201	opls_182	1	CREL	C85	85	0.14	12.011	; qtot 0.44
202	opls_185	1	CREL	H851	85	0.03	1.008	; qtot 0.47
203	opls_185	1	CREL	H852	85	0.03	1.008	; qtot 0.5
204	opls_180	1	CREL	O86	86	-0.4	15.9994	; qtot 0.1
205	opls_182	1	CREL	C87	87	0.14	12.011	; qtot 0.24
206	opls_185	1	CREL	H871	87	0.03	1.008	; qtot 0.27
207	opls_185	1	CREL	H872	87	0.03	1.008	; qtot 0.3
208	opls_182	1	CREL	C88	88	0.14	12.011	; qtot 0.44
209	opls_185	1	CREL	H881	88	0.03	1.008	; qtot 0.47
210	opls_185	1	CREL	H882	88	0.03	1.008	; qtot 0.5
211	opls_180	1	CREL	O89	89	-0.4	15.9994	; qtot 0.1
212	opls_182	1	CREL	C90	90	0.14	12.011	; qtot 0.24
213	opls_185	1	CREL	H901	90	0.03	1.008	; qtot 0.27
214	opls_185	1	CREL	H902	90	0.03	1.008	; qtot 0.3
215	opls_182	1	CREL	C91	91	0.14	12.011	; qtot 0.44
216	opls_185	1	CREL	H911	91	0.03	1.008	; qtot 0.47
217	opls_185	1	CREL	H912	91	0.03	1.008	; qtot 0.5
218	opls_180	1	CREL	O92	92	-0.4	15.9994	; qtot 0.1
219	opls_182	1	CREL	C93	93	0.14	12.011	; qtot 0.24
220	opls_185	1	CREL	H931	93	0.03	1.008	; qtot 0.27
221	opls_185	1	CREL	H932	93	0.03	1.008	; qtot 0.3
222	opls_182	1	CREL	C94	94	0.14	12.011	; qtot 0.44

223	opls_185	1	CREL	H941	94	0.03	1.008	; qtot 0.47
224	opls_185	1	CREL	H942	94	0.03	1.008	; qtot 0.5
225	opls_180	1	CREL	O95	95	-0.4	15.9994	; qtot 0.1
226	opls_182	1	CREL	C96	96	0.14	12.011	; qtot 0.24
227	opls_185	1	CREL	H961	96	0.03	1.008	; qtot 0.27
228	opls_185	1	CREL	H962	96	0.03	1.008	; qtot 0.3
229	opls_182	1	CREL	C97	97	0.14	12.011	; qtot 0.44
230	opls_185	1	CREL	H971	97	0.03	1.008	; qtot 0.47
231	opls_185	1	CREL	H972	97	0.03	1.008	; qtot 0.5
232	opls_278	1	CREL	O98	98	-0.45	15.9994	; qtot 0.05
233	opls_280	1	CREL	C99	99	0.47	12.011	; qtot 0.52
234	opls_281	1	CREL	O100	100	-0.47	15.9994	; qtot 0.05
235	opls_136	1	CREL	C101	101	-0.12	12.011	; qtot -0.07
236	opls_140	1	CREL	H1011	101	0.06	1.008	; qtot -0.01
237	opls_140	1	CREL	H1012	101	0.06	1.008	; qtot 0.05
238	opls_136	1	CREL	C102	102	-0.12	12.011	; qtot -0.07
239	opls_140	1	CREL	H1021	102	0.06	1.008	; qtot -0.01
240	opls_140	1	CREL	H1022	102	0.06	1.008	; qtot 0.05
241	opls_136	1	CREL	C103	103	-0.12	12.011	; qtot -0.07
242	opls_140	1	CREL	H1031	103	0.06	1.008	; qtot -0.01
243	opls_140	1	CREL	H1032	103	0.06	1.008	; qtot 0.05
244	opls_136	1	CREL	C104	104	-0.12	12.011	; qtot -0.07
245	opls_140	1	CREL	H1041	104	0.06	1.008	; qtot -0.01
246	opls_140	1	CREL	H1042	104	0.06	1.008	; qtot 0.05
247	opls_136	1	CREL	C105	105	-0.12	12.011	; qtot -0.07
248	opls_140	1	CREL	H1051	105	0.06	1.008	; qtot -0.01
249	opls_140	1	CREL	H1052	105	0.06	1.008	; qtot 0.05
250	opls_136	1	CREL	C106	106	-0.12	12.011	; qtot -0.07
251	opls_140	1	CREL	H1061	106	0.06	1.008	; qtot -0.01
252	opls_140	1	CREL	H1062	106	0.06	1.008	; qtot 0.05
253	opls_142	1	CREL	C107	107	-0.115	12.011	; qtot -0.065
254	opls_144	1	CREL	H107	107	0.115	1.008	; qtot 0.05
255	opls_142	1	CREL	C108	108	-0.115	12.011	; qtot -0.065
256	opls_144	1	CREL	H108	108	0.115	1.008	; qtot 0.05
257	opls_136	1	CREL	C109	109	-0.12	12.011	; qtot -0.07
258	opls_140	1	CREL	H1091	109	0.06	1.008	; qtot -0.01
259	opls_140	1	CREL	H1092	109	0.06	1.008	; qtot 0.05
260	opls_136	1	CREL	C110	110	-0.12	12.011	; qtot -0.07
261	opls_140	1	CREL	H1101	110	0.06	1.008	; qtot -0.01
262	opls_140	1	CREL	H1102	110	0.06	1.008	; qtot 0.05
263	opls_137	1	CREL	C111	111	-0.06	12.011	; qtot -0.01
264	opls_140	1	CREL	H0111	111	0.06	1.008	; qtot 0.05
265	opls_434	1	CREL	O112	112	-0.6	15.9994	; qtot -0.55
266	opls_435	1	CREL	H0112	112	0.6	1.008	; qtot 0.05
267	opls_136	1	CREL	C113	113	-0.12	12.011	; qtot -0.07
268	opls_140	1	CREL	H1131	113	0.06	1.008	; qtot -0.01
269	opls_140	1	CREL	H1132	113	0.06	1.008	; qtot 0.05
270	opls_136	1	CREL	C114	114	-0.12	12.011	; qtot -0.07
271	opls_140	1	CREL	H1141	114	0.06	1.008	; qtot -0.01
272	opls_140	1	CREL	H1142	114	0.06	1.008	; qtot 0.05
273	opls_136	1	CREL	C115	115	-0.12	12.011	; qtot -0.07
274	opls_140	1	CREL	H1151	115	0.06	1.008	; qtot -0.01
275	opls_140	1	CREL	H1152	115	0.06	1.008	; qtot 0.05
276	opls_136	1	CREL	C116	116	-0.12	12.011	; qtot -0.07
277	opls_140	1	CREL	H1161	116	0.06	1.008	; qtot -0.01
278	opls_140	1	CREL	H1162	116	0.06	1.008	; qtot 0.05
279	opls_136	1	CREL	C117	117	-0.12	12.011	; qtot -0.07

280	opls_140	1	CREL	H1171	117	0.06	1.008	; qtot -0.01
281	opls_140	1	CREL	H1172	117	0.06	1.008	; qtot 0.05
282	opls_135	1	CREL	C118	118	-0.18	12.011	; qtot -0.13
283	opls_140	1	CREL	H1181	118	0.06	1.008	; qtot -0.07
284	opls_140	1	CREL	H1182	118	0.06	1.008	; qtot -0.01
285	opls_140	1	CREL	H1183	118	0.06	1.008	; qtot 0.05
286	opls_182	1	CREL	C119	119	0.14	12.011	; qtot 0.19
287	opls_185	1	CREL	H1191	119	0.03	1.008	; qtot 0.22
288	opls_185	1	CREL	H1192	119	0.03	1.008	; qtot 0.25
289	opls_182	1	CREL	C120	120	0.14	12.011	; qtot 0.39
290	opls_185	1	CREL	H1201	120	0.03	1.008	; qtot 0.42
291	opls_185	1	CREL	H1202	120	0.03	1.008	; qtot 0.45
292	opls_180	1	CREL	O121	121	-0.4	15.9994	; qtot 0.05
293	opls_182	1	CREL	C122	122	0.14	12.011	; qtot 0.19
294	opls_185	1	CREL	H1221	122	0.03	1.008	; qtot 0.22
295	opls_185	1	CREL	H1222	122	0.03	1.008	; qtot 0.25
296	opls_182	1	CREL	C123	123	0.14	12.011	; qtot 0.39
297	opls_185	1	CREL	H1231	123	0.03	1.008	; qtot 0.42
298	opls_185	1	CREL	H1232	123	0.03	1.008	; qtot 0.45
299	opls_180	1	CREL	O124	124	-0.4	15.9994	; qtot 0.05
300	opls_182	1	CREL	C125	125	0.14	12.011	; qtot 0.19
301	opls_185	1	CREL	H1251	125	0.03	1.008	; qtot 0.22
302	opls_185	1	CREL	H1252	125	0.03	1.008	; qtot 0.25
303	opls_182	1	CREL	C126	126	0.14	12.011	; qtot 0.39
304	opls_185	1	CREL	H1261	126	0.03	1.008	; qtot 0.42
305	opls_185	1	CREL	H1262	126	0.03	1.008	; qtot 0.45
306	opls_180	1	CREL	O127	127	-0.4	15.9994	; qtot 0.05
307	opls_182	1	CREL	C128	128	0.14	12.011	; qtot 0.19
308	opls_185	1	CREL	H1281	128	0.03	1.008	; qtot 0.22
309	opls_185	1	CREL	H1282	128	0.03	1.008	; qtot 0.25
310	opls_182	1	CREL	C129	129	0.14	12.011	; qtot 0.39
311	opls_185	1	CREL	H1291	129	0.03	1.008	; qtot 0.42
312	opls_185	1	CREL	H1292	129	0.03	1.008	; qtot 0.45
313	opls_180	1	CREL	O130	130	-0.4	15.9994	; qtot 0.05
314	opls_182	1	CREL	C131	131	0.14	12.011	; qtot 0.19
315	opls_185	1	CREL	H1311	131	0.03	1.008	; qtot 0.22
316	opls_185	1	CREL	H1312	131	0.03	1.008	; qtot 0.25
317	opls_182	1	CREL	C132	132	0.14	12.011	; qtot 0.39
318	opls_185	1	CREL	H1321	132	0.03	1.008	; qtot 0.42
319	opls_185	1	CREL	H1322	132	0.03	1.008	; qtot 0.45
320	opls_180	1	CREL	O133	133	-0.4	15.9994	; qtot 0.05
321	opls_182	1	CREL	C134	134	0.14	12.011	; qtot 0.19
322	opls_185	1	CREL	H1341	134	0.03	1.008	; qtot 0.22
323	opls_185	1	CREL	H1342	134	0.03	1.008	; qtot 0.25
324	opls_182	1	CREL	C135	135	0.14	12.011	; qtot 0.39
325	opls_185	1	CREL	H1351	135	0.03	1.008	; qtot 0.42
326	opls_185	1	CREL	H1352	135	0.03	1.008	; qtot 0.45
327	opls_180	1	CREL	O136	136	-0.4	15.9994	; qtot 0.05
328	opls_182	1	CREL	C137	137	0.14	12.011	; qtot 0.19
329	opls_185	1	CREL	H1371	137	0.03	1.008	; qtot 0.22
330	opls_185	1	CREL	H1372	137	0.03	1.008	; qtot 0.25
331	opls_182	1	CREL	C138	138	0.14	12.011	; qtot 0.39
332	opls_185	1	CREL	H1381	138	0.03	1.008	; qtot 0.42
333	opls_185	1	CREL	H1382	138	0.03	1.008	; qtot 0.45
334	opls_180	1	CREL	O139	139	-0.4	15.9994	; qtot 0.05
335	opls_182	1	CREL	C140	140	0.14	12.011	; qtot 0.19
336	opls_185	1	CREL	H1401	140	0.03	1.008	; qtot 0.22

337	opls_185	1	CREL	H1402	140	0.03	1.008	; qtot 0.25
338	opls_182	1	CREL	C141	141	0.14	12.011	; qtot 0.39
339	opls_185	1	CREL	H1411	141	0.03	1.008	; qtot 0.42
340	opls_185	1	CREL	H1412	141	0.03	1.008	; qtot 0.45
341	opls_180	1	CREL	O142	142	-0.4	15.9994	; qtot 0.05
342	opls_182	1	CREL	C143	143	0.14	12.011	; qtot 0.19
343	opls_185	1	CREL	H1431	143	0.03	1.008	; qtot 0.22
344	opls_185	1	CREL	H1432	143	0.03	1.008	; qtot 0.25
345	opls_182	1	CREL	C144	144	0.14	12.011	; qtot 0.39
346	opls_185	1	CREL	H1441	144	0.03	1.008	; qtot 0.42
347	opls_185	1	CREL	H1442	144	0.03	1.008	; qtot 0.45
348	opls_180	1	CREL	O145	145	-0.4	15.9994	; qtot 0.05
349	opls_182	1	CREL	C146	146	0.14	12.011	; qtot 0.19
350	opls_185	1	CREL	H1461	146	0.03	1.008	; qtot 0.22
351	opls_185	1	CREL	H1462	146	0.03	1.008	; qtot 0.25
352	opls_182	1	CREL	C147	147	0.14	12.011	; qtot 0.39
353	opls_185	1	CREL	H1471	147	0.03	1.008	; qtot 0.42
354	opls_185	1	CREL	H1472	147	0.03	1.008	; qtot 0.45
355	opls_180	1	CREL	O148	148	-0.4	15.9994	; qtot 0.05
356	opls_182	1	CREL	C149	149	0.14	12.011	; qtot 0.19
357	opls_185	1	CREL	H1491	149	0.03	1.008	; qtot 0.22
358	opls_185	1	CREL	H1492	149	0.03	1.008	; qtot 0.25
359	opls_182	1	CREL	C150	150	0.14	12.011	; qtot 0.39
360	opls_185	1	CREL	H1501	150	0.03	1.008	; qtot 0.42
361	opls_185	1	CREL	H1502	150	0.03	1.008	; qtot 0.45
362	opls_180	1	CREL	O151	151	-0.4	15.9994	; qtot 0.05
363	opls_182	1	CREL	C152	152	0.14	12.011	; qtot 0.19
364	opls_185	1	CREL	H1521	152	0.03	1.008	; qtot 0.22
365	opls_185	1	CREL	H1522	152	0.03	1.008	; qtot 0.25
366	opls_182	1	CREL	C153	153	0.14	12.011	; qtot 0.39
367	opls_185	1	CREL	H1531	153	0.03	1.008	; qtot 0.42
368	opls_185	1	CREL	H1532	153	0.03	1.008	; qtot 0.45
369	opls_278	1	CREL	O154	154	-0.45	15.9994	; qtot 0
370	opls_280	1	CREL	C155	155	0.47	12.011	; qtot 0.47
371	opls_281	1	CREL	O156	156	-0.47	15.9994	; qtot 0
372	opls_136	1	CREL	C157	157	-0.12	12.011	; qtot -0.12
373	opls_140	1	CREL	H1571	157	0.06	1.008	; qtot -0.06
374	opls_140	1	CREL	H1572	157	0.06	1.008	; qtot 0
375	opls_136	1	CREL	C158	158	-0.12	12.011	; qtot -0.12
376	opls_140	1	CREL	H1581	158	0.06	1.008	; qtot -0.06
377	opls_140	1	CREL	H1582	158	0.06	1.008	; qtot 0
378	opls_136	1	CREL	C159	159	-0.12	12.011	; qtot -0.12
379	opls_140	1	CREL	H1591	159	0.06	1.008	; qtot -0.06
380	opls_140	1	CREL	H1592	159	0.06	1.008	; qtot 0
381	opls_136	1	CREL	C160	160	-0.12	12.011	; qtot -0.12
382	opls_140	1	CREL	H1601	160	0.06	1.008	; qtot -0.06
383	opls_140	1	CREL	H1602	160	0.06	1.008	; qtot 0
384	opls_136	1	CREL	C161	161	-0.12	12.011	; qtot -0.12
385	opls_140	1	CREL	H1611	161	0.06	1.008	; qtot -0.06
386	opls_140	1	CREL	H1612	161	0.06	1.008	; qtot 0
387	opls_136	1	CREL	C162	162	-0.12	12.011	; qtot -0.12
388	opls_140	1	CREL	H1621	162	0.06	1.008	; qtot -0.06
389	opls_140	1	CREL	H1622	162	0.06	1.008	; qtot 0
390	opls_142	1	CREL	C163	163	-0.115	12.011	; qtot -0.115
391	opls_144	1	CREL	H163	163	0.115	1.008	; qtot 0
392	opls_142	1	CREL	C164	164	-0.115	12.011	; qtot -0.115
393	opls_144	1	CREL	H164	164	0.115	1.008	; qtot 0

394	opls_136	1	CREL	C165	165	-0.12	12.011	; qtot -0.12
395	opls_140	1	CREL	H1651	165	0.06	1.008	; qtot -0.06
396	opls_140	1	CREL	H1652	165	0.06	1.008	; qtot 0
397	opls_136	1	CREL	C166	166	-0.12	12.011	; qtot -0.12
398	opls_140	1	CREL	H1661	166	0.06	1.008	; qtot -0.06
399	opls_140	1	CREL	H1662	166	0.06	1.008	; qtot 0
400	opls_137	1	CREL	C167	167	-0.06	12.011	; qtot -0.06
401	opls_140	1	CREL	H167	167	0.06	1.008	; qtot 0
402	opls_434	1	CREL	O168	168	-0.6	15.9994	; qtot -0.6
403	opls_435	1	CREL	H168	168	0.6	1.008	; qtot 0
404	opls_136	1	CREL	C169	169	-0.12	12.011	; qtot -0.12
405	opls_140	1	CREL	H1691	169	0.06	1.008	; qtot -0.06
406	opls_140	1	CREL	H1692	169	0.06	1.008	; qtot 0
407	opls_136	1	CREL	C170	170	-0.12	12.011	; qtot -0.12
408	opls_140	1	CREL	H1701	170	0.06	1.008	; qtot -0.06
409	opls_140	1	CREL	H1702	170	0.06	1.008	; qtot 0
410	opls_136	1	CREL	C171	171	-0.12	12.011	; qtot -0.12
411	opls_140	1	CREL	H1711	171	0.06	1.008	; qtot -0.06
412	opls_140	1	CREL	H1712	171	0.06	1.008	; qtot 0
413	opls_136	1	CREL	C172	172	-0.12	12.011	; qtot -0.12
414	opls_140	1	CREL	H1721	172	0.06	1.008	; qtot -0.06
415	opls_140	1	CREL	H1722	172	0.06	1.008	; qtot 0
416	opls_136	1	CREL	C173	173	-0.12	12.011	; qtot -0.12
417	opls_140	1	CREL	H1731	173	0.06	1.008	; qtot -0.06
418	opls_140	1	CREL	H1732	173	0.06	1.008	; qtot 0
419	opls_135	1	CREL	C174	174	-0.18	12.011	; qtot -0.18
420	opls_140	1	CREL	H1741	174	0.06	1.008	; qtot -0.12
421	opls_140	1	CREL	H1742	174	0.06	1.008	; qtot -0.06
422	opls_140	1	CREL	H1743	174	0.06	1.008	; qtot 0

; AA coordinates for molecule CREL (drug delivery research)

```
; crel_aa.gro (GROMACS), Fixed Format(i5,2a5,i5,3f8.3,3f8.4)
;rNum rName aName aNum  X        Y        Z
    1CREL   C01    1   4.195   4.560   4.672
    1CREL  H011    2   4.142   4.602   4.586
    1CREL  H012    3   4.239   4.465   4.643
    1CREL   O02    4   4.104   4.540   4.781
    1CREL   C03    5   4.305   4.655   4.716
    1CREL   H03    6   4.333   4.630   4.818
    1CREL   O04    7   4.417   4.642   4.626
    1CREL   C05    8   4.265   4.800   4.710
    1CREL  H051    9   4.238   4.827   4.608
    1CREL  H052   10   4.350   4.861   4.740
    1CREL   O06   11   4.158   4.828   4.802
    1CREL   C07   12   4.017   4.427   4.767
    1CREL  H071   13   4.033   4.359   4.850
    1CREL  H072   14   4.034   4.373   4.673
    1CREL   C08   15   3.872   4.478   4.771
    1CREL  H081   16   3.853   4.539   4.682
    1CREL  H082   17   3.858   4.537   4.861
    1CREL   O09   18   3.785   4.364   4.774
    1CREL   C10   19   3.647   4.404   4.778
    1CREL  H101   20   3.620   4.452   4.684
    1CREL  H102   21   3.630   4.474   4.860
    1CREL   C11   22   3.562   4.279   4.799
    1CREL  H111   23   3.456   4.306   4.797
    1CREL  H112   24   3.586   4.236   4.896
    1CREL   O12   25   3.592   4.185   4.695
    1CREL   C13   26   3.519   4.062   4.711
    1CREL  H131   27   3.413   4.082   4.700
    1CREL  H132   28   3.538   4.019   4.810
    1CREL   C14   29   3.564   3.963   4.603
    1CREL  H141   30   3.563   4.014   4.507
    1CREL  H142   31   3.495   3.879   4.601
    1CREL   O15   32   3.696   3.915   4.633
    1CREL   C16   33   3.750   3.839   4.523
    1CREL  H161   34   3.819   3.901   4.467
    1CREL  H162   35   3.671   3.804   4.456
    1CREL   C17   36   3.824   3.715   4.576
    1CREL  H171   37   3.896   3.745   4.652
    1CREL  H172   38   3.877   3.669   4.492
    1CREL   O18   39   3.727   3.625   4.632
    1CREL   C19   40   3.762   3.486   4.614
    1CREL  H191   41   3.782   3.466   4.508
    1CREL  H192   42   3.677   3.426   4.644
    1CREL   C20   43   3.883   3.443   4.698
    1CREL  H201   44   3.860   3.460   4.804
    1CREL  H202   45   3.970   3.501   4.670
    1CREL   O21   46   3.907   3.304   4.674
    1CREL   C22   47   4.008   3.251   4.761
    1CREL  H221   48   3.968   3.243   4.862
    1CREL  H222   49   4.096   3.316   4.762
    1CREL   C23   50   4.049   3.113   4.710
    1CREL  H231   51   3.960   3.050   4.707
    1CREL  H232   52   4.122   3.069   4.778
```

1CREL	O24	53	4.105	3.126	4.579
1CREL	C25	54	4.131	2.998	4.519
1CREL	H251	55	4.041	2.936	4.523
1CREL	H252	56	4.212	2.948	4.572
1CREL	C26	57	4.170	3.018	4.373
1CREL	H261	58	4.194	2.922	4.328
1CREL	H262	59	4.257	3.084	4.368
1CREL	O27	60	4.059	3.077	4.303
1CREL	C28	61	4.097	3.129	4.175
1CREL	H281	62	4.158	3.056	4.121
1CREL	H282	63	4.155	3.221	4.188
1CREL	C29	64	3.971	3.158	4.093
1CREL	H291	65	3.919	3.063	4.078
1CREL	H292	66	4.000	3.199	3.995
1CREL	O30	67	3.887	3.252	4.161
1CREL	C31	68	3.753	3.246	4.109
1CREL	H311	69	3.709	3.149	4.133
1CREL	H312	70	3.754	3.259	4.000
1CREL	C32	71	3.666	3.356	4.171
1CREL	H321	72	3.668	3.345	4.280
1CREL	H322	73	3.563	3.343	4.135
1CREL	O33	74	3.714	3.485	4.132
1CREL	C34	75	3.637	3.589	4.195
1CREL	H341	76	3.642	3.580	4.304
1CREL	H342	77	3.532	3.582	4.163
1CREL	C35	78	3.692	3.725	4.153
1CREL	H351	79	3.636	3.804	4.204
1CREL	H352	80	3.678	3.735	4.046
1CREL	O36	81	3.831	3.735	4.187
1CREL	C37	82	3.891	3.848	4.120
1CREL	H371	83	3.841	3.940	4.151
1CREL	H372	84	3.883	3.836	4.012
1CREL	C38	85	4.039	3.855	4.158
1CREL	H381	86	4.085	3.938	4.103
1CREL	H382	87	4.087	3.761	4.129
1CREL	O39	88	4.051	3.877	4.299
1CREL	C40	89	4.189	3.885	4.338
1CREL	H401	90	4.236	3.971	4.290
1CREL	H402	91	4.241	3.794	4.306
1CREL	C41	92	4.200	3.896	4.490
1CREL	H411	93	4.306	3.896	4.518
1CREL	H412	94	4.153	3.808	4.534
1CREL	O42	95	4.136	4.013	4.541
1CREL	C43	96	4.176	4.137	4.508
1CREL	O44	97	4.266	4.190	4.571
1CREL	C45	98	4.099	4.218	4.407
1CREL	H451	99	4.140	4.199	4.308
1CREL	H452	100	4.113	4.324	4.430
1CREL	C46	101	3.949	4.184	4.406
1CREL	H461	102	3.917	4.157	4.507
1CREL	H462	103	3.931	4.099	4.340
1CREL	C47	104	3.867	4.304	4.358
1CREL	H471	105	3.879	4.316	4.251
1CREL	H472	106	3.902	4.394	4.409
1CREL	C48	107	3.719	4.285	4.393
1CREL	H481	108	3.711	4.274	4.501
1CREL	H482	109	3.681	4.195	4.345

1CREL	C49	110	3.640	4.409	4.350
1CREL	H491	111	3.649	4.423	4.242
1CREL	H492	112	3.684	4.495	4.400
1CREL	C50	113	3.492	4.401	4.390
1CREL	H501	114	3.453	4.502	4.392
1CREL	H502	115	3.485	4.360	4.491
1CREL	C51	116	3.402	4.318	4.298
1CREL	H51	117	3.295	4.320	4.319
1CREL	C52	118	3.449	4.235	4.181
1CREL	H52	119	3.556	4.234	4.161
1CREL	C53	120	3.378	4.160	4.100
1CREL	H531	121	3.374	4.212	4.004
1CREL	H532	122	3.276	4.156	4.140
1CREL	C54	123	3.425	4.019	4.074
1CREL	H541	124	3.432	3.965	4.169
1CREL	H542	125	3.525	4.027	4.031
1CREL	C55	126	3.332	3.945	3.977
1CREL	H55	127	3.308	4.010	3.893
1CREL	O56	128	3.211	3.910	4.045
1CREL	H56	129	3.154	4.000	4.069
1CREL	C57	130	3.394	3.815	3.924
1CREL	H571	131	3.318	3.764	3.864
1CREL	H572	132	3.422	3.751	4.007
1CREL	C58	133	3.516	3.840	3.834
1CREL	H581	134	3.598	3.878	3.895
1CREL	H582	135	3.491	3.914	3.758
1CREL	C59	136	3.558	3.710	3.766
1CREL	H591	137	3.498	3.696	3.677
1CREL	H592	138	3.541	3.626	3.834
1CREL	C60	139	3.706	3.713	3.729
1CREL	H601	140	3.767	3.719	3.819
1CREL	H602	141	3.726	3.801	3.667
1CREL	C61	142	3.743	3.587	3.651
1CREL	H611	143	3.683	3.582	3.560
1CREL	H612	144	3.722	3.499	3.712
1CREL	C62	145	3.892	3.587	3.613
1CREL	H621	146	3.918	3.683	3.568
1CREL	H622	147	3.952	3.571	3.702
1CREL	H623	148	3.911	3.507	3.541
1CREL	C63	149	4.535	4.580	4.681
1CREL	H631	150	4.535	4.581	4.790
1CREL	H632	151	4.622	4.634	4.645
1CREL	C64	152	4.544	4.435	4.633
1CREL	H641	153	4.549	4.433	4.524
1CREL	H642	154	4.456	4.379	4.667
1CREL	O65	155	4.662	4.379	4.690
1CREL	C66	156	4.681	4.244	4.645
1CREL	H661	157	4.716	4.244	4.542
1CREL	H662	158	4.587	4.188	4.652
1CREL	C67	159	4.784	4.178	4.736
1CREL	H671	160	4.757	4.196	4.840
1CREL	H672	161	4.883	4.220	4.716
1CREL	O68	162	4.782	4.037	4.709
1CREL	C69	163	4.878	3.969	4.791
1CREL	H691	164	4.853	3.982	4.896
1CREL	H692	165	4.978	4.009	4.772
1CREL	C70	166	4.877	3.820	4.757

1CREL	H701	167	4.783	3.776	4.790
1CREL	H702	168	4.960	3.771	4.808
1CREL	O71	169	4.887	3.804	4.614
1CREL	C72	170	4.924	3.669	4.579
1CREL	H721	171	4.860	3.598	4.631
1CREL	H722	172	5.028	3.653	4.607
1CREL	C73	173	4.909	3.648	4.428
1CREL	H731	174	4.803	3.635	4.405
1CREL	H732	175	4.963	3.557	4.400
1CREL	O74	176	4.961	3.761	4.356
1CREL	C75	177	4.965	3.741	4.213
1CREL	H751	178	5.025	3.653	4.189
1CREL	H752	179	5.014	3.829	4.169
1CREL	C76	180	4.826	3.727	4.149
1CREL	H761	181	4.791	3.625	4.166
1CREL	H762	182	4.836	3.743	4.041
1CREL	O77	183	4.735	3.823	4.204
1CREL	C78	184	4.599	3.807	4.157
1CREL	H781	185	4.597	3.795	4.049
1CREL	H782	186	4.546	3.898	4.183
1CREL	C79	187	4.527	3.691	4.226
1CREL	H791	188	4.419	3.707	4.217
1CREL	H792	189	4.554	3.690	4.332
1CREL	O80	190	4.558	3.565	4.165
1CREL	C81	191	4.515	3.457	4.249
1CREL	H811	192	4.410	3.470	4.278
1CREL	H812	193	4.578	3.453	4.338
1CREL	C82	194	4.528	3.324	4.174
1CREL	H821	195	4.467	3.328	4.083
1CREL	H822	196	4.494	3.243	4.238
1CREL	O83	197	4.666	3.305	4.140
1CREL	C84	198	4.684	3.187	4.060
1CREL	H841	199	4.638	3.201	3.962
1CREL	H842	200	4.639	3.101	4.110
1CREL	C85	201	4.834	3.162	4.043
1CREL	H851	202	4.883	3.253	4.010
1CREL	H852	203	4.848	3.083	3.969
1CREL	O86	204	4.886	3.121	4.170
1CREL	C87	205	5.016	3.060	4.155
1CREL	H871	206	5.084	3.129	4.105
1CREL	H872	207	5.006	2.969	4.097
1CREL	C88	208	5.072	3.026	4.294
1CREL	H881	209	5.164	2.968	4.280
1CREL	H882	210	4.999	2.967	4.349
1CREL	O89	211	5.101	3.148	4.364
1CREL	C90	212	5.172	3.122	4.486
1CREL	H901	213	5.269	3.077	4.463
1CREL	H902	214	5.115	3.055	4.549
1CREL	C91	215	5.194	3.255	4.559
1CREL	H911	216	5.229	3.235	4.660
1CREL	H912	217	5.099	3.308	4.563
1CREL	O92	218	5.293	3.332	4.489
1CREL	C93	219	5.299	3.467	4.540
1CREL	H931	220	5.327	3.465	4.646
1CREL	H932	221	5.202	3.515	4.529
1CREL	C94	222	5.403	3.547	4.462
1CREL	H941	223	5.502	3.517	4.497

1CREL	H942	224	5.389	3.653	4.482
1CREL	O95	225	5.389	3.522	4.321
1CREL	C96	226	5.515	3.542	4.254
1CREL	H961	227	5.583	3.461	4.281
1CREL	H962	228	5.558	3.638	4.282
1CREL	C97	229	5.493	3.541	4.102
1CREL	H971	230	5.435	3.453	4.073
1CREL	H972	231	5.590	3.538	4.053
1CREL	O98	232	5.424	3.661	4.068
1CREL	C99	233	5.388	3.696	3.944
1CREL	O100	234	5.469	3.684	3.854
1CREL	C101	235	5.276	3.796	3.934
1CREL	H1011	236	5.282	3.861	4.022
1CREL	H1012	237	5.181	3.742	3.938
1CREL	C102	238	5.279	3.882	3.807
1CREL	H1021	239	5.254	3.819	3.722
1CREL	H1022	240	5.379	3.924	3.792
1CREL	C103	241	5.176	3.996	3.816
1CREL	H1031	242	5.085	3.958	3.862
1CREL	H1032	243	5.153	4.030	3.715
1CREL	C104	244	5.230	4.116	3.895
1CREL	H1041	245	5.303	4.168	3.833
1CREL	H1042	246	5.280	4.083	3.986
1CREL	C105	247	5.117	4.213	3.932
1CREL	H1051	248	5.046	4.219	3.849
1CREL	H1052	249	5.160	4.312	3.948
1CREL	C106	250	5.046	4.168	4.060
1CREL	H1061	251	5.117	4.168	4.142
1CREL	H1062	252	5.006	4.068	4.047
1CREL	C107	253	4.932	4.265	4.094
1CREL	H107	254	4.954	4.371	4.106
1CREL	C108	255	4.793	4.213	4.121
1CREL	H108	256	4.780	4.105	4.112
1CREL	C109	257	4.687	4.280	4.159
1CREL	H1091	258	4.708	4.387	4.144
1CREL	H1092	259	4.677	4.264	4.266
1CREL	C110	260	4.554	4.251	4.094
1CREL	H1101	261	4.529	4.146	4.105
1CREL	H1102	262	4.564	4.273	3.987
1CREL	C111	263	4.442	4.339	4.152
1CREL	H0111	264	4.475	4.443	4.149
1CREL	O112	265	4.415	4.302	4.288
1CREL	H0112	266	4.482	4.221	4.320
1CREL	C113	267	4.315	4.325	4.069
1CREL	H1131	268	4.269	4.227	4.086
1CREL	H1132	269	4.340	4.333	3.963
1CREL	C114	270	4.215	4.436	4.102
1CREL	H1141	271	4.261	4.533	4.080
1CREL	H1142	272	4.189	4.432	4.208
1CREL	C115	273	4.088	4.420	4.018
1CREL	H1151	274	4.035	4.330	4.049
1CREL	H1152	275	4.117	4.410	3.913
1CREL	C116	276	3.995	4.541	4.032
1CREL	H1161	277	4.050	4.632	4.004
1CREL	H1162	278	3.961	4.550	4.135
1CREL	C117	279	3.876	4.524	3.938
1CREL	H1171	280	3.822	4.432	3.965

1CREL	H1172	281	3.911	4.515	3.835
1CREL	C118	282	3.779	4.642	3.946
1CREL	H1181	283	3.694	4.624	3.879
1CREL	H1182	284	3.742	4.653	4.048
1CREL	H1183	285	3.829	4.734	3.915
1CREL	C119	286	4.044	4.889	4.740
1CREL	H1191	287	4.000	4.821	4.666
1CREL	H1192	288	4.074	4.982	4.691
1CREL	C120	289	3.941	4.920	4.848
1CREL	H1201	290	3.989	4.979	4.926
1CREL	H1202	291	3.902	4.827	4.890
1CREL	O121	292	3.834	4.996	4.788
1CREL	C122	293	3.742	5.041	4.889
1CREL	H1221	294	3.796	5.088	4.971
1CREL	H1222	295	3.685	4.957	4.927
1CREL	C123	296	3.646	5.144	4.827
1CREL	H1231	297	3.584	5.187	4.906
1CREL	H1232	298	3.581	5.092	4.757
1CREL	O124	299	3.714	5.249	4.756
1CREL	C125	300	3.800	5.329	4.839
1CREL	H1251	301	3.888	5.272	4.868
1CREL	H1252	302	3.747	5.362	4.928
1CREL	C126	303	3.846	5.452	4.760
1CREL	H1261	304	3.912	5.512	4.824
1CREL	H1262	305	3.760	5.511	4.730
1CREL	O127	306	3.918	5.407	4.645
1CREL	C128	307	3.977	5.515	4.572
1CREL	H1281	308	4.045	5.572	4.637
1CREL	H1282	309	3.900	5.582	4.533
1CREL	C129	310	4.058	5.456	4.457
1CREL	H1291	311	4.112	5.535	4.405
1CREL	H1292	312	3.988	5.409	4.387
1CREL	O130	313	4.149	5.357	4.509
1CREL	C131	314	4.209	5.282	4.402
1CREL	H1311	315	4.265	5.349	4.337
1CREL	H1312	316	4.132	5.231	4.345
1CREL	C132	317	4.304	5.177	4.461
1CREL	H1321	318	4.346	5.118	4.379
1CREL	H1322	319	4.248	5.112	4.528
1CREL	O133	320	4.410	5.243	4.531
1CREL	C134	321	4.500	5.151	4.593
1CREL	H1341	322	4.541	5.082	4.519
1CREL	H1342	323	4.448	5.094	4.671
1CREL	C135	324	4.614	5.230	4.657
1CREL	H1351	325	4.666	5.286	4.579
1CREL	H1352	326	4.684	5.162	4.706
1CREL	O136	327	4.558	5.321	4.753
1CREL	C137	328	4.658	5.409	4.806
1CREL	H1371	329	4.704	5.467	4.726
1CREL	H1372	330	4.734	5.352	4.859
1CREL	C138	331	4.590	5.505	4.905
1CREL	H1381	332	4.519	5.567	4.850
1CREL	H1382	333	4.666	5.568	4.950
1CREL	O139	334	4.522	5.429	5.006
1CREL	C140	335	4.450	5.515	5.095
1CREL	H1401	336	4.376	5.573	5.039
1CREL	H1402	337	4.519	5.583	5.146

```
1CREL  C141  338   4.378   5.429   5.199
1CREL H1411  339   4.313   5.358   5.148
1CREL H1412  340   4.317   5.494   5.262
1CREL  O142  341   4.474   5.358   5.279
1CREL  C143  342   4.409   5.284   5.383
1CREL H1431  343   4.332   5.219   5.340
1CREL H1432  344   4.363   5.351   5.455
1CREL  C144  345   4.512   5.196   5.455
1CREL H1441  346   4.558   5.129   5.381
1CREL H1442  347   4.462   5.136   5.531
1CREL  O145  348   4.612   5.278   5.516
1CREL  C146  349   4.720   5.199   5.567
1CREL H1461  350   4.758   5.132   5.489
1CREL H1462  351   4.686   5.139   5.652
1CREL  C147  352   4.835   5.290   5.613
1CREL H1471  353   4.919   5.228   5.643
1CREL H1472  354   4.802   5.350   5.698
1CREL  O148  355   4.872   5.377   5.504
1CREL  C149  356   4.991   5.451   5.536
1CREL H1491  357   5.077   5.385   5.533
1CREL H1492  358   4.983   5.496   5.636
1CREL  C150  359   5.010   5.563   5.434
1CREL H1501  360   5.010   5.520   5.334
1CREL H1502  361   5.106   5.612   5.452
1CREL  O151  362   4.904   5.660   5.446
1CREL  C152  363   4.918   5.762   5.345
1CREL H1521  364   4.921   5.716   5.247
1CREL H1522  365   5.010   5.818   5.362
1CREL  C153  366   4.799   5.857   5.351
1CREL H1531  367   4.801   5.914   5.444
1CREL H1532  368   4.706   5.799   5.347
1CREL  O154  369   4.807   5.949   5.240
1CREL  C155  370   4.712   5.954   5.144
1CREL  O156  371   4.627   6.039   5.151
1CREL  C157  372   4.714   5.858   5.028
1CREL H1571  373   4.640   5.889   4.954
1CREL H1572  374   4.686   5.761   5.067
1CREL  C158  375   4.852   5.846   4.962
1CREL H1581  376   4.849   5.761   4.894
1CREL H1582  377   4.927   5.826   5.038
1CREL  C159  378   4.891   5.971   4.883
1CREL H1591  379   4.884   6.059   4.946
1CREL H1592  380   4.823   5.983   4.798
1CREL  C160  381   5.035   5.957   4.832
1CREL H1601  382   5.048   5.860   4.784
1CREL H1602  383   5.103   5.965   4.917
1CREL  C161  384   5.066   6.067   4.730
1CREL H1611  385   5.039   6.164   4.772
1CREL H1612  386   5.008   6.050   4.640
1CREL  C162  387   5.215   6.068   4.695
1CREL H1621  388   5.245   5.970   4.657
1CREL H1622  389   5.274   6.091   4.784
1CREL  C163  390   5.241   6.175   4.588
1CREL  H163  391   5.159   6.237   4.552
1CREL  C164  392   5.381   6.198   4.536
1CREL  H164  393   5.458   6.133   4.578
1CREL  C165  394   5.423   6.288   4.451
```

1CREL	H1651	395	5.501	6.344	4.503
1CREL	H1652	396	5.341	6.357	4.430
1CREL	C166	397	5.478	6.242	4.317
1CREL	H1661	398	5.397	6.241	4.244
1CREL	H1662	399	5.517	6.141	4.328
1CREL	C167	400	5.592	6.333	4.268
1CREL	H167	401	5.671	6.332	4.344
1CREL	O168	402	5.546	6.467	4.248
1CREL	H168	403	5.518	6.511	4.344
1CREL	C169	404	5.649	6.277	4.137
1CREL	H1691	405	5.577	6.293	4.056
1CREL	H1692	406	5.666	6.170	4.149
1CREL	C170	407	5.782	6.345	4.103
1CREL	H1701	408	5.848	6.341	4.189
1CREL	H1702	409	5.763	6.449	4.076
1CREL	C171	410	5.848	6.272	3.986
1CREL	H1711	411	5.781	6.273	3.900
1CREL	H1712	412	5.868	6.169	4.015
1CREL	C172	413	5.980	6.341	3.947
1CREL	H1721	414	6.046	6.342	4.034
1CREL	H1722	415	5.960	6.443	3.915
1CREL	C173	416	6.046	6.263	3.833
1CREL	H1731	417	5.979	6.260	3.747
1CREL	H1732	418	6.067	6.161	3.866
1CREL	C174	419	6.178	6.329	3.792
1CREL	H1741	420	6.158	6.428	3.749
1CREL	H1742	421	6.243	6.340	3.879
1CREL	H1743	422	6.228	6.267	3.718
