# Supplementary material for: Drug delivery process simulation—Quantifying the conformation dynamics of paclitaxel and cremophor EL
Source: PLoS One. 2025 May 12;20(5):e0313813. doi: 10.1371/journal.pone.0313813 (PMC12068633; doi:10.1371/journal.pone.0313813)
Supplement: Attachment — Submitted filename: Response_to_Reviewers.pdf [file pone.0313813.s006.pdf]

Response to Reviewers Comments

The authors wish to again thank the editor and reviewer for very thoughtful comments. We have made every effort to respond to the issues they have raised.

Additional Editor Comments:

The discussion section can be improved by incorporating recent studies characterizing the dynamic behavior of Paclitaxel and Cremophor EL

We have added the following paragraph under ***Section (Drug Delivery Conformation Summary).***

(lines 841-850)

"More recently, research and clinical trials have focused on alternate nanoparticle formulations other than Taxol to deliver paclitaxel. Each comes with their own drug delivery characteristics and issues. All studies, or at least the grand majority, compare their delivery characteristics with those of Taxol. A good review of such studies has been presented by Sofias et al. [55]. Here they emphasize the growing acceptance of Abraxane (a 130 nm albumin-based nanoparticle) as a viable Taxol alternative. An example study comparing such behaviours has been conducted by Chen et al. [56]. Other nanoparticle formulations for paclitaxel (including liposomes) are referenced by Sofias et al. [55]. Finally, these comparisons have recently been further extended to carriers for drugs other than paclitaxel, as reviewed by Spada et al. [57], again using Taxol as a basis for comparison. Our methods should be applicable to these systems as well."

Further, in ***Section (Taxol Micelle Simulation Setup****), we added the following sentence with a reference to our recently published paper in the Journal of Physical Chemistry. This now gave a solid founadtion on the dynamic behavior of Paclitaxel and Cremophor EL*

(lines 347-348)

"In our earlier studies [51], this computational approach was first applied to setup CG network for protein molecular structure."

The following new articles are now added to the reference list, accordingly.

51. Uddin M., Coombe D. A Coarse-Grained Simulation Approach for Protein Molecular Conformation Dynamics. Journal of Physical Chemistry. 2025 Jan 16;129(2):607-622.

55. Sofias A, Dunn M, Storm G, Allen C. The battle of "nano" paclitaxel. *Adv. Drug Delivery Rev.*, 2017, v122, p20-30.

56. Chen N, Brachmann C, Liu X, Pierce D., Dey J, Kerwin W, et al. Albumin-based nanoparticle (nab) paclitaxel exhibits enhanced paclitaxel tissue distribution and tumor penetration. *Cancer Chemother. Pharmacol.*, 2015, v76, p699-712.

57. Spada A, Emami J, Tuszynski J, Lavasanifar A. The uniqueness of albumin as a carrier in nanodrug delivery. *Mol. Pharmaceutics.*, 2021, v18, p1862-1894.

Reviewer #1: The manuscript has sufficient details to be considered for publication in PLOS One, provided that the following comment is properly addressed:
The RMSD (Root Mean Square Deviation) and Radius of Gyration plots versus time must be analyzed and discussed in depth.

We have evaluated the RMSD and Radius of Gyration over the entire simulation time. Results from Table 1 show Rg (micelle of CrEL and PTX) has reached an equilibrium state at 800, 900, 1000 ns and no time plots are necessary. We have now provided in depth analysis for the RMSD and Rg. For RMSD versus time, we have now analyzed behaviour using two statistical methods.

The following paragraph is added at the end of ***Section (CrEL Aggregation Dynamics).***

(lines 627-638)

"In order to quantify the evolution of the Taxol micelle, we have analyzed the root mean square deviation (RMSD) and the radius of gyration (Rg), progressively, at every 20ns over the entire simulation period of 1000ns. Table 1 showed no noticeable changes in Rg values when other variables (such as mean square displacement, radial distribution function) also described the Taxol micelle equilibrium state. To obtain a concrete evolution picture, we have analyzed Pearson and Spearman's coefficients for RMSD for CrEL and PTX. These correlation coefficients showed a significant large positive relationship and nearly stabilized around simulation time of 1000ns with Pearson correlation of $r_p = 0.973$ (Pearson) for CrEL and $r_p = 0.834$ (Pearson) for PTX. Analogously, the Spearman rank correlation coefficients of $r_s = 0.970$ (Spearman) for CrEL and $r_s = 0.770$ (Spearman) for PTX were found. The S3 Table in S1 text (as supplementary material) presents the complete evolution of Pearson

and Spearman correlation coefficients. This study shows the utility of these two variables (RMSD, Rg) to quantify Taxol micelle dynamics.“

We have added the following new supplementary ***Section “RMSD analysis” in S1 text (as supplementary materials).***

“

RMSD Analysis

To understand Taxol micelle evolution, we have calculated Pearson correlation and Spearman’s rank correlation coefficients on RMSD data over the entire simulation period. Here we have added a sample of these analyses for clarification (S3 Table). These correlation coefficients showed a significant large positive relationship.

S3 Table. Pearson and Spearman Correlation Coefficients.

RMSD *(nm)* vs Times *(ns)* **(X-series vs Y-series)**	Pearson Correlation Coefficients		Spearman’s Rank Correlation Coefficients	
	r_p (CrEL)	r_p (PTX)	r_s (CrEL)	r_s (PTX)
800-860	0.920	0.807	0.894	0.790
860-900	0.955	0.820	0.906	0.798
900-960	0.972	0.840	0.971	0.823
960-1000	0.973	0.834	0.970	0.770

“
